# Decoding Vascular Senescence: Cellular Insights and Therapeutic Strategies

**DOI:** 10.7150/ijbs.133661

**Published:** 2026-09-02

**Authors:** Mengyuan Gao, Chongjin Zhong, Jiajia Shen, Sainan Peng, Xue Zhou, Qiuhong Shen, Cheng Qian, Xinming Yang, Yuxuan Gong, Kang Zhang, Yin Lu, Xiaodong Shu, Yang Zhao

**Affiliations:** 1 Jiangsu Key Laboratory for Pharmacology and Safety Research of Chinese Materia Medica, Nanjing University of Chinese Medicine, Nanjing 210023, China.; 2 School of Medicine, Nanjing University of Chinese Medicine, Nanjing 210023, China.; 3 Department of Gastroenterology, Taikang Xianlin Drum Tower Hospital, Affiliated Hospital of Medical School, Nanjing University, Nanjing 210046, China.

**Keywords:** vascular aging, cellular senescence, hallmarks of aging, early vascular aging (EVA), supernormal vascular aging (SUPERNOVA), senotherapeutics

## Abstract

Vascular aging is a dynamic biological process contributing to systemic organismal decay. This review elucidates the heterogeneous cellular phenotypes and microenvironmental remodeling across macrovascular and microvascular beds. These multifaceted pathological shifts are integrated into a unified tripartite framework of twelve hallmarks of vascular aging. These specific molecular dimensions ultimately dictate the clinical divergence between early vascular aging (EVA) and the supernormal vascular aging (SUPERNOVA) phenotype. Furthermore, vascular aging serves as a primary key driver of distant organ dysfunction. Based on these mechanistic insights, we summarize current behavioral and pharmacological senotherapeutic strategies targeting aged blood vessels. This systematic analysis provides critical benchmarks for deciphering vascular senescence and promoting healthy longevity.

## Introduction

In the 17th century, the English surgeon Thomas Sydenham proposed that "A man ages as much as his arteries"^1^. Currently, aging of the vascular system is gaining increasing attention in biomedical research. As a conduits of blood flow, blood vessels are constantly exposed to various systemic and localized detrimental factors^2^. Chronic exposure to these stressors induces structural and functional alterations in the vessel walls^2^. The persistent accumulation of minor cellular stress accelerates the senescence of vessels. A comprehensive understanding of these intricate regulatory mechanisms is essential for developing novel therapeutic approaches to attenuate vascular decay. Notably, vascular tissues are one of the earliest components exhibiting senescent signatures in the body^3^. These localized senescent changes sequentially trigger widespread structural abnormalities in distant non-vascular tissues^3^. Therefore, senescent vascular cells pave the path toward systemic biological aging. These specific cell subpopulations represent highly promising targets to alleviate aging-related diseases and reduce morbidity in the elderly^4^.

In this review, we comprehensively examine the localized phenotypic transitions and cell-type-specific variations within aging blood vessels. We first summarize the distinct cellular and microenvironmental alterations across different vascular beds. Subsequently, we provide a detailed evaluation of the twelve hallmarks of vascular aging, organized systematically into three functional dimensions. The documented regulatory mechanisms underlying these hallmarks allow us to characterize the physiological transition from health to disease. To bridge the gap between bench and bedside, we delineate how these molecular alterations govern the systemic divergence between the Early Vascular Aging (EVA) and the Supernormal Vascular Aging (SUPERNOVA) phenotypes. Moreover, we map the precise pathological connections linking vascular senescence to distant organ disturbances. On the basis of these findings, we review current promising interventions targeting aged blood vessels. Finally, we discuss the translational bottlenecks of current therapeutics and propose potential avenues for future research.

## Senescence in vascular aging

The vascular system, an intricate network spanning the entire human body, serves as the primary conduit for nutrient delivery, gas exchange, and metabolic waste removal^2^. With advancing age, the vascular wall undergoes a series of degenerative transitions that compromise these vital functions, laying the pathological groundwork for systemic organ decay^5^. The arterial wall is composed of three anatomical layers: the tunica intima, a single layer of endothelial cells (ECs); the tunica media, composed of multiple layers of vascular smooth muscle cells (VSMCs) interspersed with elastic fibers; and the tunica adventitia, containing adipocytes, fibrous connective tissue, and extracellular matrix (ECM) 6. Although each layer has distinct physiological properties, their collective integrity relies on a delicate balance between cellular homeostasis and microenvironmental stability^7^. During aging, this homeostasis is disrupted as the specialized cells within these layers—including ECs, VSMCs, and pericytes—undergo profound phenotypic and functional shifts^5^. Furthermore, exhaustion of the endothelial progenitor cells (EPCs) reservoir and the progressive remodeling of the ECM redefine the vascular niche, shifting it from a regenerative environment toward one characterized by chronic inflammation and structural decay^8^. This section delineates the specific senescence signatures of these cellular components and the evolving microenvironmental factors, providing a foundational framework for understanding the pathophysiology of the aging vasculature.

### ECs

The vascular endothelium, a monolayer lining the luminal surface, exhibits profound spatial heterogeneity^9^. Its senescent signatures manifest as distinct pathological phenotypes across vessels of different diameters^9^. In the intima of large and medium-sized arteries, EC senescence is characterized by impaired endothelium-dependent dilation (EDD), typically assessed by flow-mediated dilation (FMD) 10. This functional decay is primarily driven by reduced nitric oxide (NO) bioavailability. Age-associated oxidative stress triggers endothelial NO synthase (eNOS) uncoupling, shifting the enzyme from NO synthesis to superoxide (O₂⁻) production^10^. This molecular imbalance directly causes the disruption of vasomotor homeostasis. In clinic, FMD remains the gold standard for quantifying these impairments^11^.

In microvascular networks such as the blood-brain barrier (BBB) and retina, previous studies reported that senescence leads to the deconstruction of junctional complexes^12^. Specifically, the abnormal internalization of VE-cadherin and the fragmentation of tight junction proteins transform the sealed interface into a "leaky" barrier^12^. However, recent literature indicates that aging disrupts barrier homeostasis without necessarily increasing paracellular permeability^13^. Thus, in the early stages of aging, endothelial senescence drives the microvasculature into a "primed state", a state of heightened sensitivity to secondary insults without overt barrier disruption. In this state, the endothelium remains physically intact but becomes highly vulnerable, requiring additional stress or secondary insults to trigger actual physical leakage^13^. While these molecular shifts may precede barrier failure, they often manifest as detectable morphological changes in the microvascular architecture. Clinically, fundoscopic examination provides a non-invasive window for visualizing retinal microvascular remodeling, serving as a surrogate indicator for assessing systemic microvascular aging and associated complications^14^.

Furthermore, senescent ECs undergo a profound phenotypic drift known as endothelial-to-mesenchymal transition (EndoMT)^15^, wherein they lose their original endothelial identity and adopt a pro-inflammatory senescence-associated secretory phenotype (SASP). This transition not only disrupts local vascular integrity but also contributes to accelerated pathological remodeling, such as atherosclerosis^15^ and tissue fibrosis. Crucially, aging is characterized by a systemic insufficiency in VEGF signaling, which severely compromises the pro-angiogenic potential of the endothelium^1^. The resulting exhaustion of angiogenic sprouting capacity, coupled with the structural decay of existing vessels, culminates in microvascular rarefaction. This progressive loss of vessel density impairs tissue perfusion and creates a chronic hypoxic environment, ultimately driving age-related organ dysfunction.

### VSMCs

VSMCs are fundamental for governing vasomotor tone. They maintain hemodynamic stability through precise contraction-relaxation cycles^16^. Senescent VSMCs exhibit prominent morphological irregularities, characterized by the accumulation of dysfunctional organelles including the endoplasmic reticulum, Golgi apparatus, and free ribosomes^17^. A hallmark of VSMC aging is phenotypic switching, wherein cells transition from a contractile state to a multipotential, dysfunctional phenotype—encompassing calcific (osteogenic and chondrocytic), adipogenic, and macrophage-like identities^18^. This "identity crisis" is accompanied by the downregulation of contractile markers^9^, such as α-SMA, calponin, as well as an enhanced capacity for proliferation and migration from the media to the intima^19^.

This cellular transformation fundamentally reshapes the mechanobiological properties of the vessel wall. Senescent VSMCs exhibit reduced active tension, which serves as a maladaptive response to counteract the age-related increase in wall shear stress^20^. Evidence from isolated mouse aortic segments shows that aortic pressure-diameter hysteresis is attenuated by age, primarily due to the diminished contractile capacity of senescent VSMCs^21^. The disorganized spatial arrangement of VSMCs and the failure of their dynamic coupling with the ECM collectively accelerate vascular stiffening. In humans, the progressive depletion of VSMCs is substituted by disorganized collagen fibers within the medial layer^20^. Evidence from murine models suggests that this contraction-dependent compliant hysteresis is significantly mediated by the mineralocorticoid receptor (MR) in VSMCs^22^. Age-associated upregulation of MR signaling facilitates structural stiffening and fibrosis, whereas MR deficiency has been shown to attenuate these impairments^22^.

Beyond structural stiffening, VSMC senescence drives vascular calcification^16^. Senescent VSMCs within the medial layer transdifferentiate into osteoblast-like cells, secreting matrix proteins that eventually mineralize through matrix vesicle secretion, apoptosis, or progressive fibrosis^9^. Notably, VSMCs also differentiate into foam-cell-like and macrophage-like cells during the aging process, directly accelerating the progression of atherosclerotic plaques^23^. In clinical practice, these cellular and structural transitions manifest as increased arterial stiffness, which is precisely quantified via pulse wave velocity (PWV)^14^. Ultimately, this complex phenotypic remodeling underscores why VSMC senescence is a central driver of arterial aging.

### Pericytes

Pericytes reside around capillaries, precapillary arterioles, and postcapillary venules, where they maintain microvascular stability and BBB integrity. By governing vasomotor tone at the capillary level, pericytes precisely regulate regional blood flow to meet metabolic demands^24^. Recent whole-brain imaging studies have uncovered that aging triggers extensive cerebrovascular network remodeling, characterized by reduced branching and disrupted topological connectivity, leading to regional perfusion deficits^25^. Age-dependent pericyte loss not only results in reduced cerebral blood flow (CBF) and chronic hypoxia but also drives BBB breakdown, allowing the accumulation of neurotoxic plasma proteins and macromolecules, which ultimately induces secondary neurodegeneration^26^. RGS5, a specific marker of pericytes, undergoes a significant age-associated downregulation^27^. In the cerebral microvasculature, this manifests as network remodeling and BBB dysfunction; while in systemic aging, it mediates capillary rarefaction and chronic perfusion stress^28^. Conversely, during tumor angiogenesis or acute hypoxic stress, RGS5 may exhibit pathological overexpression, modulating vascular maturation via the RGS5-TGFβ signaling axis^29^. This divergent expression profile underscores the complex regulatory logic of pericytes in vascular homeostasis.

### EPCs

EPCs are essential for vascular homeostasis, displaying the dual capability to self-renew and differentiate into mature ECs^30^. Based on their kinetic and functional profiles, EPCs are categorized into two distinct subtypes: early-growth EPCs (e-EPCs), which act as circulating angiogenic cells that repair damaged ECs and modulate the vascular network via paracrine signaling, and late-growth EPCs (l-EPCs), which directly augment angiogenesis by differentiating into mature ECs^31^. EPCs function as an endogenous defense mechanism that maintains vascular integrity, but this protective efficacy is progressively compromised with age^32^. In elderly subjects, e-EPCs exhibit reduced homing capacity and functional alterations associated with endothelial dysfunction^33^. Furthermore, l-EPCs display divergent expression of genes involved in angiogenesis, coagulation, inflammation, apoptosis, and cell adhesion^34^. While EPCs in the young vasculature provide a robust reparative shield that actively retards atherogenesis, cells from aged or inflammatory environments lose this capacity and may instead undergo osteogenic differentiation, actively contributing to arterial biomineralization and stiffness^33^. This age-related functional decay is directly correlated to the failing regenerative capacity of the vascular wall; for instance, aging hinders the ability of resident cells to transition into a "progenitor-like" proliferative state following injury, thereby obstructing effective vascular repair^35^. The exhaustion and pathological shift of the EPC-mediated repair system serve as a critical driver of chronic vascular injury and subsequent systemic decay.

### Vascular microenvironment

Vascular senescence is characterized by SASP, wherein senescent cells secrete a range of pro-inflammatory cytokines, chemokines, growth factors, and matrix remodeling factors (including IL-6, IL-8, TNF-α, CXCL1, and MMP-1)^36^. SASP is a complex secretome comprising proteases, bioactive lipids, extracellular vesicles (EVs), and metabolites that collectively modulate both the local microenvironment^37^. Beyond enforcing autocrine proliferative arrest, SASP exerts profound non-cell-autonomous effects, enabling senescent cells to induce senescence in neighboring cells and recruit immune cells^38^. In parallel, vascular aging also gives rise to an array of alterations in the immune system, including the presence of increased T-lymphocytes in the vascular tissues and circulation^39^. The number of circulating CD8^+^CD28^-^ T-cells in the blood is significantly increased with aging^40^. In clinic, these inflammatory shifts are reflected in the positive correlation between red cell distribution width and systemic expression levels of SASP^32^.

ECM also plays a pivotal role in maintaining the structural integrity of the blood vessel wall, enabling it to withstand a wide range of tensile stresses. ECM is composed of abundant quantities of elastic fibers, collagen, hyaluronic acid, and proteoglycans, which provide essential durability and structural support for the blood vessels^8^. Aging gives rise to the prominent deterioration of elastic fibers in the ECM, resulting in augmented stiffness of the blood vessels accompanied by excessive accumulation of collagen^17^. Furthermore, circulating signals, particularly exosomal microRNAs, act as critical mediators of microenvironmental communication. High levels of miR-34a in the blood of elderly patients suggest its potential as an important marker of aging^41^. As specialized vehicles for autocrine and paracrine signaling, exosomes influence vascular physiological functions by delivering molecular cargo to neighboring or distant cells, thereby propagating senescence signals throughout the vascular network 42.

In summary, vascular aging is a dynamic process driven by the functional exhaustion and maladaptive transformation of diverse vascular cells, alongside the progressive deterioration of the microenvironment. These integrated alterations lead to the characteristic structural remodeling and functional decay of the senescent vasculature (Figure 1).

## The hallmarks that induce the onset of vascular aging

Advancements in gerontology have identified twelve hallmarks of aging. These hallmarks meet three criteria: they manifest during chronological aging, their experimental induction accelerates aging, and their therapeutic mitigation retards the aging process^43^. The cell-type-specific aging signatures documented in the preceding section, which encompass impaired EDD in macrovascular ECs, phenotypic switching and osteogenic transdifferentiation of VSMCs, age-dependent pericyte rarefaction, and progressive EPC exhaustion, represent the integrated downstream consequences of these converging molecular lesions. Elucidating the mechanistic basis of these phenotypic transitions requires a transition from descriptive cellular pathology to a systematic characterization of the upstream regulatory drivers.

Consequently, the following section integrates the unique structural and functional demands of the vasculature with the established gerontological framework to delineate twelve distinct hallmarks of vascular aging. These twelve hallmarks are categorized into three interrelated dimensions. The first dimension is genomic and homeostatic instability, which represents the primary molecular drivers, including genomic instability, telomere damage, epigenetic changes, and loss of protein homeostasis. The second dimension encompasses autophagy dysregulation, dysregulated nutrient sensing, and mitochondrial dysfunction, reflecting derangements at the cellular and metabolic level. The third dimension includes cellular senescence, chronic inflammation, stem cell depletion, altered intercellular communication, and impaired mechanosignaling, driving alterations at the tissue microenvironment and systemic level. Herein, we comprehensively outline these twelve hallmarks to elucidate their mechanistic underpinnings and sequential crosstalk. By systematically characterizing these twelve mechanistically defined regulatory axes, this framework directly links upstream molecular determinants to the multifaceted pathological phenotypes of vascular aging.

### Genomic and homeostatic instability

Genomic and homeostatic instability serve as the primary molecular triggers for the vascular aging program. These hallmarks include genomic instability, telomere attrition, epigenetic alterations, and loss of protein homeostasis. As upstream drivers, they compromise genomic integrity and proteomic balance. These early molecular lesions ultimately set the stage for subsequent metabolic derangement and systemic structural remodeling of the vessel wall.

#### Genomic instability

Genomic instability, driven by the cumulative DNA lesions and declined repair capacity, emerges as a primary initiator of vascular aging in both humans and mice^2^. Evidence from aging human arterial samples reveals a marked increase in DNA damage markers, which correlates significantly with early vascular aging and subclinical atherosclerosis. A major contributor to this instability is the impairment of specialized repair pathways^44^. For instance, mice deficient in nucleotide excision repair (NER) genes, such as Ercc1 or Xpd, exhibit accelerated vascular aging phenotypes, including systemic endothelial dysfunction and increased arterial stiffness^45^. Strikingly, NER deficiency in ECs leads to diminished NO bioavailability, which directly manifests as impaired EDD in clinical settings^46^. Nevertheless, the translational validity of these severe, germline DNA-repair-deficient models remains under active debate, as they may not fully recapitulate the insidious, low-dose, and chronologically driven genomic stress characteristic of natural human vascular aging. In VSMCs, persistent activation of the DNA damage response (DDR), often exacerbated by the loss of nuclear envelope proteins such as Nesprin-2, disrupts the compartmentalization of ERK1/2 signaling^47^. This signaling dysregulation impairs efficient DNA repair and promotes VSMC senescence. Furthermore, observations in human atherosclerotic lesions unraveled that unresolved DNA damage facilitates VSMC loss and fibrous cap thinning, thereby increasing plaque vulnerability^48^. Interestingly, the emergence of these phenotypes typically aligns with chronological aging, suggesting that the induction of senescence and subsequent vascular dysfunction requires a temporal threshold for DNA mutations and damage to reach a critical burden. It is noteworthy that while equivalent systemic genomic stress eventually triggers a collective vascular breakdown, neighboring ECs and VSMCs cross these threshold barriers via distinct molecular routes that predominantly drive apoptosis and phenotypic switching, respectively. Parsing these spatial and cell-specific aging thresholds remains essential for fully understanding the temporal kinetics of arterial decay.

#### Telomere damage

Telomere dysfunction, manifesting as progressive attrition or structural "uncapping", represents another critical form of genomic instability. Clinical evidence highlights a positive correlation between age-related telomere uncapping and elevated systolic blood pressure^49^. Similarly, whole-brain irradiation in mice provokes premature microvascular senescence. Within the vascular wall, both ECs and VSMCs possess low telomerase activity, resulting in progressive telomere shortening with each cell division^44^. Notably, this dysfunction is not uniformly distributed. Studies on human arterial specimens reveal more pronounced telomere impairment in ECs than in VSMCs, particularly within atheroprone regions under disturbed blood flow^50^. Mechanistically, localized telomere damage reduces NO bioavailability and triggers senescent phenotypes, subsequently facilitating immune cell recruitment after vascular injury^51^.

Importantly, substantial clinical evidence and epidemiological data demonstrate that systemic leukocyte telomere length (LTL) does not reliably correlate with preclinical or early-stage atherosclerosis^52^. This phenomenon indicates that systemic telomere shortening cannot serve as a direct predictive indicator of initial localized vascular lesions. Instead, accelerated systemic telomere attrition is more accurately defined as a cumulative, longitudinal biomarker that reflects the driving effects of shared cardiovascular stressors, such as chronic inflammation and oxidative stress, on overall biological aging^52^, ^53^. Systemic telomere loss may primarily dictate the clinical timing of advanced cardiovascular events by driving the exhaustion of hematopoietic stem cells and EPCs, which ultimately impairs plaque stabilization and endothelial turnover^52^. Consequently, a comprehensive assessment of the role of telomeres in vascular aging requires simultaneous consideration of systemic attrition kinetics and localized, lesion-specific structural uncapping characteristics.

#### Epigenetic changes

Epigenetic modifications, encompassing DNA methylation, histone modifications, and non-coding RNA (ncRNA) regulation, orchestrate gene expression patterns critical to vascular aging. DNA methylation dictates the phenotypic switching of diverse vascular cells^54^. Specifically, dysregulation of key regulators, such as DNMTs and METTL14, drives endothelial dysfunction and VSMC phenotypic transition^54^, ^55^; simultaneously, DNA methylation alterations also modulate the activation of vascular fibroblasts and macrophages^54^. Human cohort studies have demonstrated that early-life cardiovascular health is "imprinted" on genome-wide DNA methylation patterns, serving as a stable indicator of midlife arterial stiffness and subclinical pathology^56^. Additionally, the Sirtuin (SIRT) family attenuates cellular senescence across the vascular wall, yet age-related NAD^+^ depletion compromises this protection, exacerbating arterial stiffness, systolic and diastolic dysfunction, and aortic remodeling^57^. Conversely, classical HDACs such as HDAC5 and HDAC3 drive vascular hypertrophy and VSMC senescence by mediating vasoconstriction, oxidative stress, and histone lactylation, thereby accelerating systemic vascular aging^58^, ^59^. ncRNAs also represent extensively investigated epigenetic factors in vascular aging. MicroRNA-21 and microRNA-34a both regulate VSMC phenotypic switching and act as emerging biomarkers for arterial senescence^60^, ^61^. Nevertheless, current research focuses predominantly on microRNA. The functional significance of other ncRNAs remains controversial and necessitates further clinical and preclinical validation^62^.

#### Loss of protein homeostasis

Intracellular protein homeostasis is frequently disrupted by the increased accumulation of mistranslated or misfolded proteins, a primary driver of vascular aging 63. Aging has significant impacts on various components of the protein homeostasis system in the vascular system, encompassing macroautophagy, the ubiquitin-proteasome system, as well as the chaperone-mediated autophagy (CMA)^64^. Specifically, CMA emerges as a selective defense mechanism against vascular aging by degrading pro-atherogenic proteins and maintaining VSMC proteostasis^63^, while clearing inflamed protein complexes (such as NLRC4/NLRP3) to restrain endovascular inflammation^65^. Remarkably, the progressive failure of CMA, marked by diminished expression of its key receptor LAMP2A, not only serves as a functional indicator of proteostatic collapse, but also acts as a reliable pathological biomarker of human plaque instability^65^. As vascular aging advances, this decline in CMA and molecular chaperones (such as HSP70) triggers an escalating accumulation of cytotoxic protein aggregates and inflammasomes, which in turn amplifies oxidative stress and accelerates atherogenesis in a self-propagating feed-forward loop^64^. Although non-invasive tracking of autophagic flux in clinical settings remains challenging, reviving chaperone-mediated protein quality control offers a viable avenue to forestall vascular senescence and halt the progression of arterial remodeling.

### Cellular and metabolic dysfunction

Metabolic derangement represents a critical transition in the vascular aging program, translating upstream genomic and homeostatic instability into sustained functional decay. The erosion of proteostatic balance and genomic integrity inevitably cascades into the collapse of nutrient-sensing networks and the accumulation of damaged organelles. Within the vasculature, this metabolic shift is primarily manifested through the dysregulation of autophagic dysregulation, dysregulated nutrient sensing, and mitochondrial dysfunction. These interrelated metabolic hallmarks fail to operate in isolation but rather form a self-perpetuating cycle that amplifies oxidative stress and inflammatory signaling. By converting early molecular lesions into chronic energetic failure and pro-oxidant states, these metabolic disturbances act as the proximal drivers for the systemic structural remodeling and clinical manifestations of the aging vasculature.

#### Autophagy dysregulation

Autophagic activity tends to diminish with aging, contributing to the buildup of damaged proteins and organelles during the aging process. Within the vasculature, the expression of core autophagy genes, such as ATG5 and ATG7, is significantly decreased over time^66^. In VSMCs, the impairment of ATG7-dependent autophagy confers to premature senescence, characterized by nuclear hypertrophy, contractile dysfunction, excessive collagen deposition, and accelerated atherogenesis^66^. Similarly, the loss of ATG7 in ECs leads to abnormal intracellular lipid accumulation and compromised vessel integrity, thereby accelerating atherogenesis^67^. Beyond maintaining general proteostasis, specialized autophagic pathways including mitophagy are essential for preserving endothelial integrity. In aging coronary arteries, the failure of FUNDC1-dependent mitophagy results in the accumulation of dysfunctional mitochondria, which elevates oxidative stress and augments endothelial senescence^68^. Ultimately, the collapse of the autophagic system acts as a pivotal factor in converting early molecular lesions into chronic inflammation and systemic structural remodeling of the arterial wall^69^. Nevertheless, current mechanistic evidence remains primarily restricted to endothelial autophagy, with limited insights into how autophagic decline manifests in other critical vascular cell types, such as VSMCs and pericytes. Furthermore, as most studies rely on acute gene knockout models, whether such total gene inactivation authentically reflects the subtle, chronic decline of autophagic flux during human physiological aging remains to be fully verified.

#### Nutritional sensing disorder

The nutrient-sensing network comprising evolutionarily conserved pathways including mTOR, AMPK, and SIRTs functions as a metabolic command center that orchestrates cellular responses to nutrient availability and growth signals. In vascular aging, mTOR is a critical driver of functional decay. Hyperactivation of mTOR signaling, particularly mTORC1, promotes the translation of IL-1α to fuel the SASP^70^. Extensive evidence demonstrates that pharmacological or genetic inhibition of mTOR can delay endothelial senescence and modulate the phenotypic switching of VSMCs, thereby mitigating vascular stiffness as well as oxidative stress^71^. Notably, mTOR dysregulation has been deemed to be a pivotal regulator linking cerebrovascular dysfunction to neurodegenerative conditions such as Alzheimer's disease and atherosclerosis^72^. The energy-sensing axis of SIRTs and AMPK undergoes a progressive decay with age^73^. SIRTs, which are strictly dependent on NAD^+^ availability, exert a beneficial effect on healthy aging by virtue of increasing resistance to DNA damage and metabolic disruption^74^. Similarly, AMPK functions as a primary energy sensor that markedly enhances endothelial function, augment NO bioavailability, and attenuate oxidative stress^73^. Together, the imbalance between nutrient-driven growth and energy-driven maintenance may be a fundamental metabolic driver of systemic vascular aging. Despite the established benefits of modulating individual pathways, a fundamental challenge remains in how the vascular wall orchestrates the crosstalk between hyperactivated mTOR and depleted AMPK/SIRT signaling, as non-selectively targeting single nodes may inadvertently disturb basal metabolic homeostasis.

#### Mitochondrial dysfunction

Mitochondria, regarded as cellular powerhouses, play an important role in modulating the aging process. As individuals age, oxidative stress within the vascular system is exacerbated by the elevated generation of ROS in the absence of sufficient antioxidant defenses, driving vascular senescence phenotypes including endothelial dysfunction and atherosclerosis^75^. Recent data have demonstrated that production of mitochondrial ROS (mtROS) plays a pivotal role in aggravating these dysfunctions^76^. In aging blood vessels, particularly in regions with disrupted flow (pedicles, branches, and bifurcations), there is a significant surge in the production of intramitochondrial ROS originating from the electron transport chain, xanthine oxidase, as well as NADPH oxidase. Uncoupled eNOS is capable of generating ROS, which interferes with NO to produce peroxynitrite, thereby diminishing the bioavailability of NO, impairing endothelium-dependent dilation and triggering a pro-oxidant phenotype in aged ECs^77^. Moreover, vascular aging is closely associated with impaired mitochondrial energy metabolism and the depletion of NAD^+^, a conserved characteristic of aging across species^78^. Nevertheless, NAD^+^ has dual effects. On one hand, the accumulation of DNA damage and mitochondrial dysfunction provoked by low NAD^+^ levels exacerbate the progression of senescence. On the other hand, the exacerbation of SASP seems to be highly metabolically demanding, and the age-related decay in NAD^+^ may paradoxically act as a metabolic constraint that limit the progression of SASP^78^. Additionally, the age-dependent accumulation of mitochondrial DNA mutations and deletions further erodes mitochondrial energy production, thereby establishing a self-perpetuating cycle that accelerates the aging process.

### Cellular instability and microenvironmental evolution

The accumulation of molecular and metabolic damage ultimately leads to the exhaustion of cellular regenerative capacity and the deterioration of the vascular microenvironment. These systemic alterations, characterized by permanent cell cycle arrest and dysregulated intercellular signaling, transform the vasculature into a pro-inflammatory and dysfunctional tissue environment. By driving the progression from intracellular molecular defects to multicellular functional decay, these hallmarks establish the mechanistic basis for arterial remodeling and age-related vascular pathologies.

#### Senescent cell accumulation

In the process of aging in humans, senescent cells tend to accumulate at divergent rates in numerous tissues. Cellular senescence is defined as a terminal state of cell cycle arrest characterized by the irreversible loss of proliferative capacity^79^. This program is primarily executed through the p53/p21^WAF1/Cip1^ and p16^INK4A^/pRB axes, which inhibit cyclin-dependent kinases to prevent E2F-mediated DNA replication^80^. While initially a tumor-suppressive mechanism, the excessive accumulation of senescent cells drives vascular aging^80^. In elderly individuals, p53, p21, and p16 levels are significantly elevated in the endothelium, an effect that is attenuated by regular physical activity^81^. Mechanistically, p53 hyperactivation drives capillary rarefaction, whereas its downstream effector p21 (encoded by Cdkn1a) functions as a complicated regulator of vascular homeostasis^82^. Moderate p21 expression is essential for maintaining the quiescence of EPCs and supporting angiogenesis^82^. Furthermore, p16-INK4A (encoded by Cdkn2a) serves as a definitive marker of the senescent state. Although the clearance of p16-positive cells has been shown to delay age-associated disorders, subsequent studies have revealed that age-induced p16 accumulation is a gradual process and that the majority of p16-high cells are vascular ECs^83^. Importantly, certain p16-high subpopulations, such as liver sinusoidal ECs are indispensable for maintaining tissue healthspan^83^. To address this functional complexity, an *in vivo* genetic toolbox was generated, consisting of three p16-related intersectional genetic systems^84^. This toolset allows for the precise tracking, ablation, and manipulation of p16-positive cells *in vivo*, enabling the identification of cell-type-specific functional roles for senescence across diverse tissues^84^. Ultimately, the progressive accumulation of these senescent populations results in a systemic decay in vascular compliance and the functional collapse of capillary networks. These findings suggest that cellular senescence is functionally heterogeneous rather than uniformly detrimental. Consequently, future senolytic strategies likely require cell type- and tissue-specific targeting to preserve the beneficial functions of selected senescent populations while eliminating pathogenic ones.

#### Chronic inflammation

Chronic inflammation is recognized as a persistent driving force that spans the entire progression of vascular aging^43^. Diverse extrinsic and intrinsic stressors promote the acquisition of SASP by inducing DNA damage and suppressing endogenous repair mechanisms^85^. During the progression of vascular senescence, the inflammatory environment is sustained through autocrine and paracrine signaling loops^86^. Pro-inflammatory genes are upregulated in ECs and VSMCs, resulting in the elevated production of chemokines, adhesion molecules, and cytokines (IL-1β, IL-6, TNF-α, etc.)^2^, ^43^. The loss of endogenous regulatory proteins, such as Annexin A1, aggravates this inflammatory state and accelerates the transition toward a pro-senescent phenotype^87^. In clinical conditions of premature aging, such as Takayasu's arteritis, the synergy between VSMC senescence and chronic inflammation establishes a self-perpetuating cycle of vascular injury^88^. In the advanced stages, the chronic elevation of pro-inflammatory molecules is positively associated with vascular sclerosis and negatively correlated with endothelium-dependent vasodilation. Age-associated increases in vascular permeability facilitate the infiltration of circulating immune cells into the vascular wall^89^. These cells release additional cytokines, transforming localized cellular stress into a systemic inflammatory state^43^. Ultimately, this inflammatory milieu impairs angiogenic capacity and leads to the collapse of microvascular homeostasis^90^. Although anti-inflammatory strategies mitigate vascular damage, broad immunosuppression may inadvertently compromise essential host defense mechanisms and tissue repair pathways.

#### Stem cell depletion

Unlike the functional impairment of circulating progenitors discussed previously, stem cell exhaustion refers to the numerical contraction and loss of self-renewal potential within the primitive stem cell pools, such as vascular wall-resident stem cells and bone marrow-derived mesenchymal stem cells (MSCs) 91. The maintenance of the vasculature relies on the ability of these stem cells to remain in a quiescent state and undergo controlled activation upon injury^92^. However, aging disrupts this homeostatic balance through a synergy of extrinsic and intrinsic stressors. Long-term exposure to SASP, coupled with intrinsic genomic instability and mitochondrial dysfunction, forces quiescent stem cells into premature exhaustion or permanent senescence^93^. This multifaceted decay effectively compromises the "stemness" of the regenerative reservoir. Specifically, aged MSCs exhibit a significantly diminished capacity to promote angiogenesis and support endothelial function, thereby failing to maintain the vascular niche^94^. Furthermore, recent evidence suggests that stem cell exhaustion is accompanied by a qualitative lineage dysregulation. During aging, hematopoietic stem cells undergo myeloid-biased differentiation, which tilts the systemic environment toward a pro-inflammatory state and accelerates vascular endothelial damage^95^. Overall, the exhaustion and maladaptive transformation of the stem cell pool serve as a crucial driver of vascular aging, marking the failure of the vessel's endogenous regenerative capacity.

#### Altered intercellular communication

Beyond cell-intrinsic defects, aging triggers profound alterations in intercellular communication^43^. These changes orchestrate the systemic decay of the vascular microenvironment and overall vascular function. This dysregulation is primarily mediated through SASP. SASP factors facilitate the horizontal transmission of senescence to neighboring healthy cells, a process known as paracrine senescence^2^. In recent years, EVs have become critical modulators of intercellular and interorgan communications. Aging significantly alters the cargo of EVs, including specific proteins and microRNAs^96^. These altered EVs act as potent vehicles for vascular pathology. Specifically, senescent cell-derived EVs accelerate the accumulation of pathologic amyloid in the ECM^96^. Under stress conditions, such as endoplasmic reticulum stress, VSMCs increase the secretion of EVs enriched with glucose-modulated proteins and aggravate vascular calcification. Furthermore, EV-encapsulated miR-21 induces senescence and impairs the angiogenic potential of recipient ECs^97^. Beyond vesicular transport, direct cell-to-cell communication is also impaired. The disruption of Notch signaling and gap junction connectivity decouples the functional coordination between ECs and VSMCs^6^. These systemic shifts in the vascular secretome and communication network ensure that localized cellular stress is amplified into widespread tissue dysfunction, thereby propelling the progression of vascular aging.

#### Impaired mechanosignaling

Building upon the structural alterations in vascular stiffness, the impairment of mechanosignaling represents a fundamental hallmark where physical cues are aberrantly translated into pro-senescent biochemical response^6^. Vascular cells, particularly ECs and VSMCs, rely on sophisticated mechanosensors such as integrins and Piezo1 to perceive hemodynamic shear stress and tensile strain^98^. During aging, the progressive remodeling and stiffening of the ECM disrupt these sensory mechanisms, contributing to the chronic activation of maladaptive signaling pathways. Central to this process is the dysregulation of the YAP/TAZ transcriptional co-activators^99^. In a youthful, compliant vascular environment, balanced mechanical forces maintain YAP/TAZ activity to support cellular homeostasis. However, the increased stiffness of the aged vascular wall forces the persistent nuclear translocation of YAP/TAZ, which paradoxically triggers pro-inflammatory gene expression and accelerates vascular senescence^100^. This mechanical-biochemical mismatch further promotes the transition of VSMCs from a contractile to a synthetic phenotype, exacerbating vessel wall thickening and calcification 101. Indeed, the aberrant activation of endothelial YAP/TAZ has been shown to drive the progression of atherosclerosis by amplifying localized inflammatory responses within the vascular wall^101^. In sum, the failure of cells to accurately sense and respond to their physical microenvironment emerges as a decisive driver that locks the vasculature into a self-perpetuating cycle of structural disintegration and functional decay.

### Crosstalk among cardiovascular aging hallmarks

The hallmarks of vascular aging do not operate in isolation. They form an interconnected and self-perpetuating network across molecular, cellular, and systemic levels^6^. This crosstalk ensures that localized molecular lesions are rapidly amplified throughout the vascular tissue. This process establishes a feedback loop that sustains the vascular aging.

Genomic instability and telomere attrition are regarded as the primary triggers. These factors initiate the cascade by activating DDR. Persistent DDR forces cells into permanent senescence. It also compromises mitochondrial function and protein homeostasis^47^. In this context, the depletion of NAD^+^ acts as a metabolic nexus. The decay of NAD^+^ impairs SIRT-mediated epigenetic stability and mitochondrial oxidative phosphorylation^57^. Paradoxically, low NAD^+^ levels are also prone to be a metabolic constraint that limits the intensity of the SASP^78^. Altered intercellular communication bridges the transition from cell-intrinsic damage to systemic dysfunction. SASP factors and pro-senescent EVs serve as as horizontal transmitters. They spread inflammatory signals and senescence to neighboring healthy cells and distant stem cell pools^96^. This process accelerates stem cell depletion^93^. The structural remodeling of the ECM further complicates this biochemical environment. Increased vascular stiffness disrupts mechanosignaling pathways^99^. More specifically, the aberrant nuclear translocation of YAP/TAZ fuels the production of pro-inflammatory cytokines and promotes further ECM deposition^101^

These synergistic interactions suggest that vascular aging is a non-linear, multi-dimensional cascade rather than a simple accumulation of independent defects. Multi-level biomarker profiling in elderly individuals provides clinical evidence: systemic telomere length and tissue-specific senescent cell accumulation represent distinct, parallel pathophysiological dimensions rather than a sequential cause-and-effect cascade. While systemic telomere attrition reflects intrinsic replicative limits across progenitor pools, localized senescent populations are independently driven by microenvironmental stress and SASP-mediated signaling^102^. Therefore, the traditional "one-target, one-drug" approach may be insufficient to counteract such a robust and interconnected biological program. Future investigations and therapeutic interventions must shift focus from isolated hallmarks toward the systemic nodes of crosstalk. By identifying the master regulators that link metabolic, mechanical, and inflammatory pathways, we can provide a more sophisticated theoretical basis for multitargeted rejuvenation strategies, aiming to restore the holistic homeostasis of the aging vasculature (Figure 2).

## Macro-phenotypes of vascular aging: from EVA to SUPERNOVA

### The divergent paths of vascular aging

The chronological aging of the vascular system fails to progress uniformly across individuals. Instead, it diverges into distinct macro-phenotypes representing polar extremes of arterial health. The concept of these vascular extremes allows for the stratification of patient populations based on the decoupling of biological vascular age from chronological age^103^.

When the physiological aging of the arterial wall is prematurely accelerated, it induces a phenotype designated as EVA. Pathophysiologically, EVA manifests as accelerated atherosclerosis, arteriolar sclerosis, carotid intimal-medial thickening, endothelial dysfunction, enhanced vasoconstriction of blood vessels, and increased total peripheral resistance^104^. These compounded alterations results in a premature loss of arterial compliance and compromised target organ perfusion.

In sharp contrast to the accelerated decay observed in EVA, a unique subpopulation displays a delayed vascular aging trajectory, known as SUPERNOVA^103^. Individuals harboring the SUPERNOVA phenotype maintain optimal arterial elasticity despite advancing chronological aging. Functionally, they do not develop the typical age-associated elevations in blood pressure, progressive systemic arterial stiffness, or clinical atherosclerosis^105^. Their arterial walls remain structurally resilient, preserving the compliance levels characteristic of significantly younger cohorts. As a result of this sustained vascular preservation, the SUPERNOVA group exhibits a significantly diminished risk of clinical cardiovascular events and all-cause mortality compared to chronological peers exhibiting normal or accelerated vascular aging trajectories^103^.

### Clinical matrix and stratification standards

PWV serves as the clinical gold standard for quantifying arterial stiffness and functions as an independent predictor of all-cause and cardiovascular mortality 105. The clinical determination and stratification of vascular aging extremes rely on a dynamic matrix of hemodynamic metrics, metabolic risk factors, and structural parameters. Traditional cardiovascular risk factors, including hypertension, diabetes mellitus, elevated ultrasensitive C-reactive protein (hs-CRP), and high uric acid levels, directly accelerate arterial stiffening, thereby driving the EVA phenotype 105. In contrast, the absence of these risk factors—frequently observed in females and individuals with lower resting heart rates—preserves low PWV values across lifespan cohorts, establishing the foundation for the SUPERNOVA phenotype^106^. Under this operational matrix, the stratification standards are strictly threshold-dependent and are characterized by the vascular age gap (Δ-age), which is mathematically defined as the individual's actual chronological age minus their calculated biological vascular age. Specifically, EVA is diagnosed when an individual's aortic PWV exceeds the 90th percentile of the age- and sex-matched reference population, leading to a negative Δ-age where biological vascular age exceeds chronological age^103^. Conversely, the SUPERNOVA phenotype is defined when the aortic PWV remains below the 10th percentile of the corresponding chronological peer group in the absence of clinical cardiovascular disease, yielding a prominent positive Δ-age where biological vascular age is significantly lower than chronological age^103^. Within this SUPERNOVA population, the magnitude of the positive Δ-age is a quantitative indicator of cardiovascular resilience, where the numerical value of Δ-age is inversely correlated with the incidence of adverse cardiovascular events^103^.

Methodologically, the determination of vascular age can be achieved through different diagnostic modalities. Brachial-ankle PWV (baPWV) and carotid-femoral PWV (cfPWV) both provide reliable measurements for estimating vascular age and predicting cardiovascular risk, demonstrating high consistency in clinical cohorts. Beyond one-dimensional functional metrics, advancements in three-dimensional aortic geometry utilizing magnetic resonance imaging have revealed that specific structural parameters, such as aortic arch curvature and tortuosity, possess distinct genetic determinants. These geometric alterations tightly associate with functional PWV changes and direct clinical outcomes in EVA patients^107^. Therefore, integrating functional PWV measurements with geometric modeling optimizes the clinical matrix for identifying vascular aging trajectories.

### Pathophysiological and genetic determinants of vascular extremes

The phenotypic divergence between EVA and the SUPERNOVA is determined by a complicated interplay of genetic predisposition, structural geometry, and lifelong environmental exposures rather than by isolated risk factors alone. Genetic background plays a foundational role in establishing these vascular extremes. Genome-wide analyses and clinical biobank data show that specific genetic variants dictate baseline arterial compliance and structural vulnerability^107^. These genetic determinants directly influence three-dimensional aortic geometry, where distinct inheritable traits modulate aortic arch curvature and tortuosity. In individuals prone to EVA, these genetically driven geometric alterations alter regional hemodynamic shear stress, creating a localized mechanical environment that accelerates wall remodeling and functional stiffening independently of traditional metabolic risks^107^. This mechanical maladaptation corresponds to impaired mechanosignaling, one of the core twelve hallmarks of vascular aging, which translates aberrant physical stimuli into pro-senescent vascular remodeling. Beyond genetic architecture, early life origins and cumulative systemic stressors dictate the long-term trajectory toward EVA. The EVA syndrome often originates from fetal programming, low birth weight, or early childhood metabolic insults, which permanently alter the structural scaffolding of large elastic arteries. This early structural setting creates a continuous pathological cascade. In these vulnerable vessels, systemic low-grade inflammation, another canonical vascular aging hallmark, acts as a pivotal driver, with elevated circulating cytokines and immune dysregulation serving as precursors for atherothrombosis, subclinical arterial calcification, and accelerated EVA progression^108^.

Genetic variations within the SIRT and uncoupling protein (UCP) families act as vital upstream regulatory molecules corresponding to two core metabolic hallmarks of vascular aging. Abnormal polymorphisms in SIRT genes compromise endothelial epigenetic stability and disrupt mitochondrial metabolic homeostasis^109^. This exacerbates the DNA damage response and promotes mitochondrial ROS accumulation, which ultimately amplifies SASP release and sustains the chronic inflammatory environment seen in EVA vessels. Similarly, dysfunctional UCP signaling abolishes adaptive mitochondrial uncoupling, compounding ROS production and extracellular matrix remodeling. Conversely, conserved and highly functional SIRT and UCP genetic profiles maintain stable epigenetic modifications and stabilize mitochondrial redox homeostasis^109^. This restrains excessive oxidative stress and dampens inflammatory cascades, forming the core molecular mechanism underlying the vasculoprotective effects of the SUPERNOVA phenotype. In EVA individuals, persistent impairment of these metabolic pathways synergizes with dysregulated mechanosignaling and chronic inflammation to form a self-amplifying pro-senescence loop, which accelerates irreversible vascular structural deterioration and functional decline. In contrast, sustained metabolic homeostasis mediated by intact SIRT/UCP signaling decouples chronological aging from vascular deterioration, enabling preserved arterial compliance and resisting stress-induced vascular remodeling in SUPERNOVA individuals^110^. To this end, while EVA represents a synergy of genetic vulnerability and early environmental insults, the SUPERNOVA phenotype is sustained by protective genetic factors and favorable physiological traits that insulate the arterial tree from systemic decay^103^.

Although macroscopic clinical differences between the EVA and SUPERNOVA phenotypes have been well characterized, mechanistic exploration of their phenotypic divergence remains insufficient and falls behind clinical phenotypic observations. Notably, the sex-specific predominance of the SUPERNOVA phenotype observed in epidemiological cohorts has not been fully explained at the molecular level. The interactive relationships between sex-dependent physiological factors, genetic variants, and multi-hallmark crosstalk during vascular aging remain poorly characterized^103^. Furthermore, available studies mostly focus on individual senescence-associated molecules rather than systematically dissecting the synergistic or antagonistic interactions across the twelve vascular aging hallmarks. The cell-type-specific functions of SIRT and UCP families in ECs and VSMCs also lack precise verification.

### Translational perspectives and strategies for vascular reversibility

Vascular aging extremes do not represent permanent or static clinical states. The EVA phenotype harbors distinct windows of susceptibility where the accelerated aging trajectory can be halted or partially reversed^105^. Shifting an individual's vascular path from an EVA template toward a healthy vascular aging or SUPERNOVA profile requires early lifestyle modifications and strict metabolic risk management^103^. Epidemiological and clinical data unveil that maintaining a lifestyle characterized by non-smoking, non-drinking, and regular physical activity directly correlates with lower resting heart rates, reduced plasma uric acid, and suppressed hs-CRP levels, which collectively insulate the arterial wall against progressive stiffening^103^. Furthermore, preventing or aggressively managing traditional cardiovascular risk factors, in particular hypertension and diabetes mellitus, remains mandatory to preserve optimal PWV values across shifting age cohorts^104^, ^106^.

From the perspective of clinical screening and public health prediction, phenotypic identification based on vascular age evaluation provides an effective tool for early risk stratification. Clinical assessment of Δ-age through baPWV and cfPWV detection enables precise recognition of divergent vascular aging phenotypes^103^. Tracking and maximizing this positive Δ-age provides a precise clinical approach to enhance individual cardiovascular resilience, ultimately serving as a foundational strategy for retarding vascular aging and reducing cardiovascular mortality on a population scale^103^. Beyond routine clinical screening and intervention, integrated translational research strategies targeting SUPERNOVA cohorts facilitate the exploration of core protective mechanisms underlying sustained arterial elasticity. Multi-dimensional profiling combining familial lineage analysis, multi-organ functional evaluation, high-throughput genomic detection including GWAS, exome sequencing and epigenetic modification mapping, together with high-resolution phenotypic characterization, contributes to the identification of key vascular protective regulatory factors^111^. After clarifying the core molecular pathways driving arterial accelerated senescence, screening protective variants of aging susceptibility genes in SUPERNOVA populations can help discover novel therapeutic targets, providing a feasible translational approach to replicate vascular anti-aging protective effects in general populations.

## Vascular aging and related diseases

Vascular aging operates as a pivotal pathological mechanism that drives the onset and progression of diverse systemic disorders. This aging process systematically compromises various anatomical compartments, directly accelerating the initiation of hypertension, chronic kidney disease, cardiovascular and cerebrovascular diseases. Epidemiological analyses consistently confirm that these clinical condition manifest with significantly higher incidence in the elderly than in the young population. Therefore, prioritizing research into how vascular aging dictates the pathogenesis of age-associated disorders is imperative to facilitate the development of novel therapeutic strategies that effectively ameliorate these conditions in old individuals.

### Neurodegenerative and cerebrovascular diseases

#### Alzheimer's disease (AD)

The pathogenesis and clinical progression of AD are closely interwined with age-associated microvascular alterations, challenging the traditional neurocentric view of cognitive decline. This process is driven by cell-intrinsic senescence within the neurovascular unit^112^. Recent community-based autopsy investigations have illustrated that distinct cerebrovascular pathologies are present in over 50% of clinically diagnosed AD cases, underscoring the underrecognized contribution of vascular aging to neurodegeneration^113^. Mechanistically, this vulnerability is driven by the progressive degradation of the BBB. As cerebral microvessels age, EC senescence and the chronic secretion of the SASP—particularly MMPs and pro-inflammatory cytokines—disrupt endothelial junctions and trigger the progressive loss of brain pericytes^114^. This architectural breakdown of the BBB, accompanied by basement membrane thickening and astrocytic end-feet detachment, leads to systemic leakage, allowing blood-borne neurotoxins to infiltrate the brain parenchyma while simultaneously downregulating transmembrane clearance receptors on senescent ECs^114^. This failure of receptor-mediated trans-endothelial transport obstructs the continuous clearance of amyloid-β (Aβ) from the brain parenchyma into the circulation, accelerating the formation of neurotoxic Aβ plaques and neurodegenerative cascades^115^. Large-scale multi-factorial data-driven analyses from cohorts such as the AD Neuroimaging Initiative validate that this vascular dysregulation and resulting cerebrovascular insufficiency serve as the earliest detectable biomarkers of AD trajectory, occurring decades before clinical symptomatology and driving the irreversible initiation of neurodegenerative cascades^116^.

#### Vascular cognitive impairment (VCI)

Completely distinct from the barrier-clearance failure characterizing AD, VCI represents the direct longitudinal consequence of systemic vascular aging on cerebral hemodynamics and parenchymal bioenergetics^117^. In this setting, the disease trajectory is predominantly dictated by structural vessel wall remodeling, arterial stiffness, and microvascular senescence^118^. Driven by upstream metabolic syndrome, insulin resistance, and dysregulated nutrient sensing, VSMCs undergo a phenotypic shift from a contractile to a synthetic, pro-calcifying profile, causing advanced collagen cross-linking and severe arteriolosclerosis^119^. This loss of arterial compliance—manifested in both intracranial and extracranial vessels—fails to buffer systemic pulsatile pressure, transmitting destructive mechanical shear stress into the fragile cerebral microenvironment, which fundamentally destroys CBF autoregulation^120^. Concurrently, EC senescence and impaired compensatory angiogenesis induce a progressive dying-back of capillary networks, termed microvascular rarefaction^121^. At the subcellular level, this rarefaction is heavily compounded by cerebromicrovascular mitochondrial dysfunction and excessive ROS production, which disrupts endothelial-pericyte crosstalk and drives chronic hypoperfusion^122^. This net reduction in capillary density strips the deep-penetrating subcortical white matter of essential oxygen and nutrient delivery, triggering chronic local ischemia, neuroinflammation, and axonal demyelination^118^. Neuroimaging systematic reviews highlight that these ischemic white matter hyperintensities and subcortical small vessel lesions correlate linearly with executive dysfunction, highlighting cerebromicrovascular senescence as a primary therapeutic target to halt the onset of vascular-associated dementia cascades^123^.

### Metabolic and microvascular diseases

#### Diabetes mellitus

Diabetes mellitus is a complicated metabolic disorder characterized by impaired glucose tolerance and persistent hyperglycemia^108^. Sustained exposure to hyperglycemia increases the risk of developing systemic vascular disorders. These disorders are classified into microvascular complications and macrovascular complications^124^. The primary underlying pathophysiologic mechanisms of these complications include endothelial dysfunction, accelerated arterial stiffness, systemic inflammation, and the thickening of capillary basement membrane^124^. In the diabetic environment, these pathological alterations are characterized by a form of premature vascular aging^108^. A central molecular mechanism of this premature aging is the non-enzymatic accumulation of advanced glycation end products (AGEs) in tissues. Endogenous AGEs bind directly to the receptor for advanced glycation end-products (RAGE) expressed on ECs and VSMCs^125^. The interactions between AGEs and RAGE induce the intracellular production of ROS and drives oxidative stress. This oxidative stress axis suppresses the expression of eNOS in human coronary artery ECs^125^. Concurrently, hyperglycemia and elevated ROS concentrations upregulate the expression levels of specific inflammatory and cell adhesion molecules, includingVCAM-1, ICAM-1, MCP-1, as well as E-selectin, thereby resulting in endothelium-dependent vasodilatory dysfunction and further progression of diabetes mellitus.

#### Diabetic retinopathy (DR)

DR has been regarded as one of the most common and severe microvascular complications of diabetes mellitus, in particular in the older patients^126^. Diabetes mellitus and the resulting hyperglycemia are the predominant contributors to the development of DR^126^. At the clinical level, the symptoms of DR include increased vascular permeability, the formation of microaneurysms, hard exudates, and intraretinal hemorrhages^127^. At the cellular level, the critical characteristics of DR are vascular dysfunction, retinal capillary loss, and neuroretinal degeneration. These features directly parallel the hallmarks observed in classical vascular aging^126^. Specifically, the chronic diabetic environment elicits the selective loss of retinal pericytes and EC apoptosis^127^. The degeneration of these cells alters the homeostatic interaction between ECs and pericytes, which impairs the integrity of the blood-retinal barrier (BRB)^127^. Furthermore, retinal vascular dysfunction triggers elevated expression of pro-inflammatory cytokines and angiogenic growth factors derived from various local cell types. These factors include TNF-α, IL-6, and VEGF^127^. The sustained upregulation of these pro-inflammatory cytokines and growth factors disrupts capillary junctions, increases retinal vascular permeability, and drives abnormal neovascularization, thereby accelerating the clinical progression of DR^126^.

### Hypertension and cardiovascular diseases (CVD)

#### Hypertension

Hypertension is a primary clinical manifestation of vascular aging characterized by structural remodeling of the arterial wall and mechanical dysfunction^128^. Systemic aging increases arterial stiffness by altering the molecular phenotypes of ECs and VSMCs, alongside degrading the composition of the ECM^20^. In the setting of accelerated arterial stiffness, the characteristic impedance and forward wave amplitude of aortic root are augmented^129^. This alteration causes the reflected wave to reach the heart prematurely during systole, which selectively elevates systolic blood pressure and reduces diastolic blood pressure, thereby increasing the clinical pulse pressure^129^. Atherosclerosis normally precedes and results in hypertension while hypertension tends to accelerate arterial stiffness, implying the presence of a positive feedback loop. Under the condition of hypertension, the aging-associated vascular alterations are exacerbated, contributing to extensive vascular damage. Furthermore, this loss of vascular compliance reduces the structural elasticity of the carotid artery wall in close proximity to the arterial baroreceptors^130^. This reduction in compliance can disrupt the pressure receptors, particularly during postural changes, thereby predisposing patients with vascular aging to undergo orthostatic hypotension^130^. In young hypertensive individuals, these interactions promote early vascular aging, rendering their microvascular and macrovascular phenotypes highly similar to those of old adults^128^.

#### CVD

According to epidemiological projections, CVD, particular heart diseases and stroke, serve as the leading cause of global morbidity and mortality, accounting for a high burden of annual deaths as the elderly population rises^131^. This age-related risk is dictated by a complex interplay between the molecular influences of vascular senescence and tissue-specific pathophysiological mechanisms^132^. Under the circumstance of vascular aging, the stiffness of aorta elevates, arterial compliance diminishes, and reflected waves from aorta and proximal arteries tend to reach the heart earlier. This contributes to increased left ventricular afterload and myocardial oxygen demand, thereby reducing coronary artery perfusion and blood supply^20^. These chronic alterations collectively induce left ventricular hypertrophy, myocardial ischemia, coronary artery disease, and heart failure^133^. In clinic, the profiles and manifestations of these cardiovascular events vary substantially by sex across different vascular beds. Due to sex differences in myocardial and vascular aging, non-obstructive coronary artery disease in female patients often manifests as more diffuse and extensive than obstructive coronary artery disease with an equivalent clinical symptom burden^133^.

Concurrently, macrovascular structural failures, including aortic dissection and aortic aneurysm, impose an escalating therapeutic challenge within aging populations. Investigations delineating how cell-intrinsic vascular senescence dictates the pathogenesis of each specific disease condition remain limited. Further research targeted at these distinct etiologies is vital to elucidate the unresolved molecular mechanisms, which will ultimately facilitate the development of novel therapeutic strategies for mitigating vascular aging.

### Renal and heritable vascular diseases

#### Chronic kidney disease (CKD)

CKD is characterized by aberrant renal structure and function lasting more than three months. This progressive disease exhibits a global prevalence of approximately 13.4% and imposes a severe public health burden due to its direct association with elevated cardiovascular risk^134^. CKD is thought to be a prominent public health problem owing to its correlation with high cardiovascular risk. The progression of patients with CKD is aggravated by premature vascular and cardiac aging as well as ectopic calcification^135^. Because renal blood flow accounts for approximately 20% of the total cardiac output, the renal microvasculature is highly sensitive to macrovascular hemodynamic alterations. During aging, cardiovascular calcification triggers pulse waves to longitudinally shear the endothelium along vascular routes^136^. This leads to vasodilatation, vascular remodeling, and ultimately thrombosis or embolism. These events are able to result in glomerular injury, reduced glomerular filtration rate, proteinuria, hematuria, as well as renal function impairment^136^. Crucially, pathological vascular remodeling and renal insufficiency exhibit a bidirectional relationship rather than a unidirectional cascade. Patients with advanced coronary artery calcification show an increased risk of developing incident CKD. Conversely, patients with CKD who lack diagnosed cardiovascular diseases frequently present high coronary artery calcification scores, which correlate linearly with adverse renal outcomes and the accelerated loss of kidney function^137^.

#### Hereditary diseases

Beyond acquired metabolic and hemodynamic risk factors, a distinct variety of inherited genetic conditions accelerate cell-intrinsic vascular aging and precipitate premature death^138^. Early-onset vascular aging is regarded as an important feature of various types of inherited diseases. For instance, familial hypercholesterolemia comprises a group of inherited disorders that disturb lipid metabolism and accelerate atherosclerosis, which can manifest early in life^138^. Similarly, generalized arterial calcification of infancy originates *in utero* and provoke severe stenosis, hypertension, as well as heart failure, which is frequently fatal within the first 6 months of life^139^. Hutchinson-Gilford progeria syndrome, originating from mutations in the LMNA gene^140^, simulates different aspects of vascular aging found in the elderly, such as atherosclerosis, arterial stiffness, as well as calcification. The syndrome's related defects frequently contribute to death during the second decade of life, predominantly owing to myocardial infarction or stroke^140^. Delving into the precise molecular mechanisms of these genetic models is essential for elucidating the unresolved pathways of premature vascular senescence, which will ultimately facilitate the development of novel therapeutic strategies aimed at alleviating vascular aging.

### Common pathophysiological pathways across diseases

Vascular aging typically occurs earlier than the clinical manifestations of disease and is a high-risk factor for the development of vascular aging-associated diseases^128^, ^129^. There is a reciprocal interactions between vascular aging and vascular diseases; aging blood vessels provide an environment conducive to the onset and progression of vascular diseases, while vascular diseases, in turn, accelerate the process of vascular aging^128^. Vascular aging does not function as an isolated physiological decline but rather serves as a foundational pathological matrix that drives a diverse cluster of systemic diseases (Figure 4).

The first primordial pathway is the systematic degradation of endothelial and pericyte barrier integrity, which converts tight selective barriers into architectural hyperpermeability zones^114^. Under normal physiological conditions, specialized vascular barriers dictate molecular transport; however, the age-dependent downregulation of endothelial junctional proteins, such as VE-cadherin and Claudin-5, coupled with the progressive senescence and loss of pericytes, disrupts this structural gating^114^. In the central nervous system, this barrier breakdown manifests as BBB leakage, driving neurodegenerative cascades in AD 112. In the ocular microcirculation, an identical disruption of the BRB precipitates plasma fluid and lipid exudation, clinically presenting as hard exudates and macular edema in DR^126^.

The second convergent pathway involves structural vessel wall remodeling and microvascular rarefaction, which collectively culminate in sustained tissue hypoperfusion and chronic hypoxia^118^, ^120^. In large conductive arteries, vascular aging is characterized by the phenotypic switching of VSMCs from a contractile to a synthetic profile, alongside advanced collagen deposition and matrix degradation. This structural remodeling drives arterial stiffness and eliminates hemodynamic buffering capacity, transmitting destructive pulsatile pressure and shear stress downstream to propel systemic hypertension^129^. Meanwhile, at the microcirculatory level, the exhaustion of EPCs and the uncoupling of endogenous angiogenic VEGF signaling provoke a progressive dying-back of capillary networks, termed microvascular rarefaction^121^. This net reduction in capillary density strips parenchymal tissues of essential oxygen and nutrients^120^. In the central nervous system, this microvascular rarefaction propels chronic local ischemia and small vessel lesions, accelerating VCI^118^, ^122^. Similarly, in CKD, the structural obliteration of glomerular capillaries deprives nephrons of perfusion, contributing to glomerular injury, a decreased glomerular filtration rate, and an irreversible loss of renal function^136^.

In summary, vascular aging across different organs exhibits distinct, tissue-specific molecular mechanisms due to their varied anatomical compartments. Likewise, some of these pathological processes intersect through shared, overlapping pathways, displaying varied clinical manifestations across specific diseases. Targeting these common hallmarks of vascular senescence provides an integrative therapeutic strategy to mitigate a broad spectrum of age-related systemic diseases. Therefore, future investigative efforts are encouraged to adopt a comprehensive perspective. While continuing to delineate these organ-specific pathways, more research focus should concurrently be directed toward exploring these shared, overarching mechanisms.

## Behavioral and pharmacological interventions to retard vascular aging

The lifelong accumulation of factors that cause vascular dysfunction inevitably contributes to progression of associated diseases. In this sense, therapeutic strategies should be multifaceted to address these diverse contributors. In this section, we mainly summarize a variety of behavioral and pharmacological interventions aimed at alleviating vascular aging. All of these interventions have been correlated to reduced incidence of age-associated diseases and improved in human longevity. In fact, a healthy lifestyle is a critical approach to delay and treat vascular aging. Such healthy lifestyle is composed of a balanced diet including the Mediterranean diet, appropriate exercise, smoking cessation, stress management, and calorie restriction. The effects of these practices on vascular health are listed in Table 1.

### Behavioral interventions

#### Exercise training

Clinical evidence from the Lifestyle Interventions and Independence for Elders (LIFE) study reveals that structured physical activity significantly reduces the risk of major mobility disability in vulnerable older adults^141^. Regular exercise is known to provoke benefits for health and longevity as well as to diminish the risk of cardiovascular diseases. Appropriate exercise increases circulating blood volume by approximately 20% to 25%, enhances capillary density and capillary-to-fiber ratio, as well as improves mitochondrial content and function, all of which are driven by increased oxidative capacity^142^. Exercise can also attenuate the blood pressure, boost systemic metabolism, augment insulin sensitivity, and mitigate hyperlipidemia. Regular physical activities have been demonstrated to diminish the traditional cardiovascular risk factors that are involved in the progression of AD, such as impaired blood flow^143^. In fact, short-term exercise training augments NO production and bioavailability, thereby enhancing conduit artery endothelial function, and extended training tends to yield shear stress-induced arterial remodeling^144^. Moreover, long-term aerobic exercise training is closely correlated to alteration in cardiac morphology. The underlying mechanisms of exercise on vascular protection are multifactorial, predominantly including the maintenance of NO homeostasis, reduction in formation of ROS, improved endothelial function, amelioration of arterial stiffness, as well as inhibited inflammatory response^145^.

#### Calorie restriction (CR)

CR, which involves reducing caloric intake without yielding malnutrition has been shown to slow the process of aging in organisms ranging from yeast to primates 146. However, the absolute reliability of translating lifespan extension from non-human primates to humans remains a subject of intense academic debate, as confounding husbandry and lifestyle factors highly influence survival outcomes^146^. In clinical studies, the landmark Comprehensive Assessment of Long-term Effects of Reducing Intake of Energy (CALERIE) trials uncovered that a 2-year modest human CR is highly feasible and significantly reduces biological predictors of healthspan^147^. CR ameliorates vascular senescence through elevated eNOS activity, reduced age-mediated oxidative stress, enhanced autophagy, abated degradation of aortic elastin, as well as increased expression level and activity of SIRT1 in VSMCs^148^. Long-term CR is extensively effective in diminishing the risk of atherosclerosis in humans. Moreover, CR is effective on enhancing systemic metabolic profiles as well as limiting obesity and insulin resistance^149^. Nevertheless, long-term adherence to continuous CR faces major translational hurdles. Recent clinical evaluations have demonstrated that continuous CR can cause potential neurobehavioral or sleep quality fluctuations in adults^150^. Therefore, future investigative efforts should evaluate whether optimizing macronutrient composition and intermittent fasting windows can suppress vascular aging. This tailored strategy may offer a more sustainable approach to retard arterial stiffness without compromising patient compliance.

#### The Mediterranean diet

Of note, the Mediterranean diet provides considerable health benefits resulting from its anti-inflammatory, anti-atherosclerotic, and antioxidant effects. It may play a critical role in limiting telomere shortening and is positively associated with telomere length, enabling it to be a crucial mediator of an anti-aging lifestyle^151^. In large-scale population studies, such as the EVA study, high adherence to the Mediterranean diet is strongly correlated to a higher prevalence of healthy vascular aging^152^. Clinical assessments using the Cardio-Ankle Vascular Index (CAVI) further illustrate that sustained Mediterranean dietary patterns significantly preserve arterial elasticity and restrict aging-related vascular degradation, particularly in women^153^. Furthermore, this protective phenotype remains consistent even under complex clinical conditions, showing a direct positive relationship with preserved vascular structure and functional parameters^154^. In spite of the undeniable beneficial effects of behavioral strategies on healthspan and longevity, long-term adherence to these habits is obviously challenging. Of interest, a major knowledge gap persists regarding the exact molecular crosstalk between specific Mediterranean micronutrients and EC phenotypic transitions. Future clinical trials are supposed to elucidate whether these whole-dietary benefits can be effectively recapitulated through targeted, individual nutraceutical interventions. As such, pharmacological manipulation of these specific nutrient-governed signaling pathways may offer a more practical and effective route for preventing vascular aging.

### Pharmacological interventions

In addition to healthy lifestyles, a series of pharmacologic therapies can be utilized to hamper vascular aging. Therapeutic agents traditionally employed to antagonize risk factors have been observed to enhance vascular function, diminish arterial stiffness, and retard vascular aging. Multiple new medications for managing risk factors including hypertension, hyperlipidemia, and diabetes have also exhibited the capability to mitigate arterial stiffness, repair endothelial dysfunction, as well as improve vascular remodeling. Drugs including statins, metformin, rapamycin, resveratrol, and NAD^+^ prevent vascular aging by acting on the blood vessels in multiple ways, as summarized in **Table 1**.

#### Statins

Statins can directly restore and improve vascular function by boosting the level of NO in the endothelium, enhancing reendothelialization following arterial injury, and restricting the inflammatory effects in the vessel wall 155. Clinical trials have also demonstrated that the patients receiving statins display decreased low-density lipoprotein (LDL) cholesterol and experience few cardiovascular events^155^. Atorvastatin, a type of statins, tends to mitigate the activation of NLRP3 and decrease the levels of IL-1β and IL-18 in atherosclerotic patients^156^. Repression of the pro-atherogenic signaling cascades (NLRP3 or type I interferon) may be beneficial for patients with cardiovascular diseases following the treatment of statins^156^. Recent preclinical evidence underscores that statins are inclined to mitigate stress-related vascular aging and atherosclerosis by modulating the glucagon-like peptide-1/adiponectin axis^157^. In chronic kidney disease conditions, statin therapies consistently alleviate cellular senescence and vascular degradation. Furthermore, simvastatin directly ameliorates senescence-induced mitochondrial dysfunction in VSMCs 158. Nevertheless, long-term therapy of statins is closely correlated with severe coronary artery calcification. Mechanistically, this phenomenon represents a phenotypic paradox rather than direct vascular toxicity. Statins promote a minor osteogenic trans-differentiation of VSMCs within the plaque area^158^. This microcalcification process accelerates the conversion of vulnerable lipid-rich soft plaques into stable, calcified hard plaques, thereby preventing plaque rupture and clinical ischemic events^158^. Currently, the large-scale Pragmatic Evaluation of Events and Benefits of Lipid Lowering in Older Adults trials are actively assessing the real-world clinical benefits and long-term safety of this statin-mediated remodeling in elderly cohorts^159^.

#### Metformin

Metformin is not only an antihyperglycemic agent but also exerts protective effects on the vascular endothelium. It alleviates high glucose-induced oxidative stress by hindering the activation of NADPH oxidase pathway^160^. Furthermore, metformin tends to prevent cardiovascular complications correlated with diabetes and atherosclerosis by suppressing endothelial senescence and dysfunction via the epigenetic regulation of SIRT1 and components of PRMT1^161^. Recent mechanistic analyses consistently support these protective roles, demonstrating that metformin suppresses endothelial senescence primarily via activating the AMPK signaling pathway. This activation subsequently downregulates mTOR signaling and diminishes the release of SASP^162^. Beyond ECs, metformin also suppresses VSMC senescencethrough promoting autophagic flux. In clinical settings, endothelial function has been found to be improved in the patients treated with metformin. From clinical and translational perspectives, this pleiotropic agent offers a highly promising role in delaying human vascular aging and providing comprehensive cardioprotection^163^. Additionally, despite its favorable safety profile, clinicians must carefully consider rare but severe translational risks, including metformin-associated lactic acidosis in vulnerable cohorts^160^.

#### Rapamycin

Rapamycin, a potent inhibitor for mTOR, is normally employed as an immunosuppressant in organ transplant recipients and as an anti-proliferative drug for the treatment of certain types of tumors^164^. It alleviates peripheral blood flow defects in mouse models of atherosclerosis and AD by maintaining mitochondrial function, diminishing the accumulation of Aβ, and decreasing mTOR activity^72^. Dietary rapamycin supplementation effectively reverses age-related vascular dysfunction and oxidative stress by modulating nutrient-sensing and cell cycle pathways^71^. Furthermore, rapamycin is able to reduce the arterial stiffness, blood pressure, left ventricular hypertrophy, and lower the cardiovascular risk in patients with coronary artery disease and cardiac allograft vasculopathy^165^. It also suppresses the expression level of TNF-mediated VCAM-1 and ameliorates endothelial inflammation. Concurrently, dual mTORC1/mTORC2 inhibitors, such as Palomid 529, significantly restrict tumor angiogenesis and vascular permeability^166^. Recent data from a pilot phase 1 clinical trial unraveled that while rapamycin treatment is feasible in elderly cohorts with cognitive decline, safety parameters require meticulous monitoring^167^. Therefore, future investigations must establish precise, low-dose intermittent regimens or localized delivery strategies to maximize vascular benefits without provoking systemic toxicities.

#### Resveratrol

Resveratrol, a natural plant polyphenol, has displayed prominent vasoprotective effects in various preclinical models. It retards the progression of DR by mitigating vascular leakage and loss of pericytes, and by modulating the level of VEGF^127^ . Resveratrol has also been found to diminish the number of senescent ECs, reduce the expression levels of TLR4-mediated pro-inflammatory proteins including MMP-3 and MMP-9 in the aged mice, and enhance vascular structure^168^. Furthermore, preclinical evidence demonstrates that resveratrol effectively attenuates age-related vascular remodeling by repressing the local renin-angiotensin system and downregulating angiotensin II type 1 receptor expression^169^. In silico analyses consistently validate its robust capacity to interact with longevity-associated regulatory proteins. Molecular docking and simulation data pinpoint that resveratrol exhibits exceptionally high binding affinities to stabilize the protein structures of SIRT1, SIRT6, and SREBF1^170^. A randomized controlled trial elaborated that dietary resveratrol supplementation significantly improves endothelial function and increases mitochondrial density in older adults^171^. Unlike its clear vascular and mitochondrial benefits, long-term resveratrol administration fails to modulate systemic glucose metabolism or insulin sensitivity in non-diabetic elderly populations^171^. Hence, future clinical investigative efforts must decipher this metabolic-vascular uncoupling.

#### NAD+

NAD^+^ serves as a ubiquitous metabolite involved in crucial redox reactions. It is prone to participate in glycolysis and mitochondrial oxidative phosphorylation, and modulates the balance of cellular redox through SIRT family proteins. In the process of aging, NAD^+^ levels decline in multiple tissues and cells, including vascular components^74^. Consequently, NAD^+^ supplementation is deemed to counteract with the aging process. Administration of NAD^+^ precursor, including nicotinamide (NAM), nicotinamide riboside, and nicotinamide mononucleotide (NMN), boosts NAD^+^ levels both *in vitro* and *in vivo*^172^. Exogenous NAD^+^ supply directly alleviates mitochondrial oxidative stress in ECs and rescues related vascular dysfunction^173^. However, distinct NAD^+^ precursors exhibit marked heterogeneity regarding their systemic safety and therapeutic outcomes. As a NAD^+^ precursor, NAM serve as a potent pan-SIRT inhibitors and can remarkably shorten lifespan in lower organisms^174^. Nevertheless, long-term NAM administration in the aged mice fed with high-fat diets yields a prominent reduction in inflammation and improves healthy aging without prolonging lifespan. Diverging from the limitations of NAM, NMN represents a highly reliable and safe precursor pathway. Chronic NMN supplementation is well tolerated as a long-term dietary intervention for up to 12 months in mice without inducing toxic feedback^172^. On the basis of the vascular theory of aging, impairment of blood vessels plays a critical role in driving age-associated diseases. In the process of aging, quadriceps capillary density decreases accordingly. NMN supplementation alleviates this age-associated arterial dysfunction by reducing oxidative stress and restoring SIRT1 activity^74^. This capillary rejuvenation enhances physical performance in a Sirt1- and VEGF-dependent manner^175^. Notably, short-term NMN treatment robustly rescues the diminished capillary number in the mouse quadriceps even at 32 months of age^74^. In the central nervous system, studies have shown that NMN is able to rescue BBB leakage provoked by aging or lipopolysaccharide by improving the function of mitochondria in a SIRT3-dependent manner^176^. Supplementation with NMN can normalize ROS levels generated by mitochondria and improve the endothelial function of cerebral blood vessels and the neurovascular coupling responses in aged mice^177^. To this end, NAD^+^ serves as a critical modulator in repairing vascular injuries provoked by aging and various stressors.

#### Angiotensin-converting enzyme (ACE) inhibitors

ACE inhibitors or angiotensin receptor antagonists are extensively employed for treating hypertension and heart failure. They have demonstrated striking benefits including enhanced endothelial function, resistance to collagen fibrosis, as well as reduced arterial stiffness^178^. From a broader geroscience perspective, current evidence indicates that renin-angiotensin system inhibitors positively influence multiple conserved aging regulatory pathways, highlighting their clinical potential to protect against human biological aging^178^. Meanwhile, large-scale multinational population-based cohort studies unveil that the long-term use of specific antihypertensive drug classes significantly reduces the risk of incident dementia in elderly populations^179^. Additionally, ACE inhibitors play a vital role in preventing vascular inflammation by downregulating pro-inflammatory cytokine cascades within the vessel wall^180^. However, long-term therapeutic regimens with ACE inhibitors introduce significant translational risks during clinical procedures. Patients chronically treated with these agents frequently develop refractory hypotension during general anesthesia induction^181^. Mechanistically, chronic ACE inhibition induces a profound compensatory upregulation of eNOS to maintain basal vascular tone^181^. Subsequent exposure to general anesthetics, such as propofol, hyperactivates this vascular relaxation pathway, thereby triggering catastrophic arterial dilation^181^. Future investigative efforts should define precise withdrawal kinetics for different ACE inhibitors to maximize vascular aging protection while preventing intraoperative hemodynamic collapse.

#### Prebiotics & probiotics

Gut microbiome dysbiosis results in arterial stiffness and aggravates vascular aging in aged mice, which indicates that targeting gut microbiome may provide a potential therapeutic strategy to prevent or combat age-associated arterial stiffness. Of note, *ganoderma lucidum* terpene derivative has been shown to retard obesity-related atherosclerosis through augmenting the abundance of certain gut microbes and increasing the level of branched-chain amino acid^182^. A growing body of evidence indicates that probiotics and prebiotics are able to decrease the level of trimethylamine oxide (TMAO) and alleviate the atherosclerotic lesions through remodeling the gut microbiota^183^. Nonetheless, the human gut microbiome varies significantly across different populations. Associated adverse effects also exhibit remarkable inter-individual diversity. In this regard, validating the clinical efficacy and safety of specific probiotics and prebiotics against vascular aging rigorously requires more extensive clinical cohorts with larger sample sizes.

#### Senolytics

Senolytics represent a specialized class of pharmacological agents that selectively eliminate senescent cells. Intermittent oral administration of the representative senolytic cocktails, dasatinib and quercetin, significantly decreases the burden of senescent cells within the aortic inner layer of aged mice, thereby remarkably improving vasodilatory function^184^. Mechanistically, these agents achieve selectivity by disabling distinct nodes of senescent cell anti-apoptotic pathways (SCAPs). Dasatinib predominantly inhibits ephrin receptors and tyrosine kinases, whereas quercetin targets the PI3K/AKT, p21, and BCL-2 protein families^184^. This targeted elimination holds pivotal therapeutic potential to diminish macrovascular and microvascular morbidity. However, a critical translational hurdle involves the substantial off-target toxicities on non-senescent, healthy cells. Because several nodes of SCAPs are shared by normal proliferating cells and somatic stem cells under physiological conditions, systemic senolytic administration frequently triggers severe side effects, including myelosuppression, neutropenia, and transient endothelial denudation^185^. Furthermore, ongoing clinical studies have not yet established efficacy specifically for human vascular aging conditions^186^. To overcome these safety limitations, future pharmacological strategies are supposed to pivot from systemic small-molecule deployment toward stimulus-responsive smart drug delivery systems. Utilizing galactose-encapsulated nanoparticles designed for intracellular release via senescence-associated β-galactosidase cleavage^187^, or implementing targeted CAR-T cell therapies against senescent surface antigens^188^, will be essential for achieving precise vascular rejuvenation without inducing healthy tissue destruction.

On the basis of the identification of contributors and diseases that influence the progression of vascular aging, an array of anti-aging strategies promote healthy aging and prevent the onset of associated vascular diseases. At present, behavioral approaches remain highly promising. Noteworthily, these lifestyle interventions face remarkable challenges resulting from limited long-term individual compliance with regular exercise and lifelong dietary restrictions. Alternatively, the utilization of pharmacologic strategies to delay biological aging in healthy individuals remains highly controversial. The potential long-term adverse effects of chronic systemic administration frequently surpass the therapeutic benefits. Crucially, the majority of current candidates fail to directly target endothelial senescence due to an ongoing reliance on non-specific systemic pathways. Therefore, future investigative efforts should pivot toward deciphering cell-surface markers specific to senescent ECs or VSMCs. Developing stimulus-responsive smart drug delivery systems or multi-targeted senomorphics of great significance to achieve precise vascular rejuvenation without inducing systemic homeostatic disruptions.

## Conclusion and future perspectives

Vascular aging is a highly dynamic and orchestrated biological program. Within this process, the constituent cells of blood vessels undergo distinct alterations across macrovascular and microvascular beds. This pathological progression concurrently involves specific cellular phenotypic transitions and localized microenvironmental changes. This review first systematically outlines these multifaceted shifts during vascular senescence (Figure 1). Subsequently, these recent breakthroughs are integrated into a unified tripartite framework of twelve hallmarks of vascular aging. These hallmarks encompass upstream genomic and homeostatic instability, intermediary defects in autophagy, nutrient sensing, and mitochondrial energetics, as well as downstream cellular senescence accumulation, stem cell exhaustion, chronic inflammation, and impaired mechanosignaling (Figure 2). These molecular dimensions ultimately govern distinct physiological outcomes and multi-organ clinical diseases. They dictate the systemic divergence between EVA and the newly characterized SUPERNOVA phenotype (Figure 3). Moreover, vascular aging acts as a primary catalyst for widespread organ dysfunction (Figure 4). It operates through universal pathological pathways, including BBB leakage in AD and BRB collapse in DR, as well as structural microvascular rarefaction coupled with arterial stiffening. To combat these pathological cascades, an array of anti-aging interventions has been deployed. Behavioral approaches including exercise and caloric restriction effectively retard early arterial stiffening. Pharmacological strategies further target these pathways (Table 1).

However, the field still confronts several conceptual challenges and translational bottlenecks. First, the strength of evidence supporting different hallmarks of vascular aging remains highly uneven; while robust clinical and preclinical studies lend strong support to pathways like mitochondrial oxidative stress and chronic low-grade inflammation, emerging mechanisms such as extracellular vesicle-mediated signaling are currently confined to early-stage experimental settings. Second, the direct causal relationships between early-stage molecular insults and downstream cellular senescence or macroscopic vascular tissue remodeling remain difficult to define. Furthermore, the current diagnostics of EVA and SUPERNOVA rely heavily on arbitrary, cohort-specific percentiles of the vascular age gap (Δ-age). This clinically-derived phenotypic stratification is currently disconnected from mechanistic preclinical validation, underscoring an urgent need to explicitly integrate the SUPERNOVA resilient phenotype with the molecular hallmarks of aging.

Beyond these conceptual gaps, practical implementation of senotherapeutic interventions faces additional hurdles. Standardized dietary supplements and systemic small-molecule deployment often exhibit variable efficacy due to limited tissue specificity. Existing preclinical research still relies heavily on simplified, isolated EC cultures. This paradigm fails to recapitulate the complex, multi-layered spatial architecture of native blood vessels *in vivo*. Therefore, future investigative efforts look to pivot toward precision geroscience and advanced bioengineering. First, dissecting the precise genetic and metabolic determinants protecting the SUPERNOVA population warrants prioritized investigation. Second, developing multi-cellular, biomimetic platforms such as vascularized organs-on-a-chip serves as a viable path to establish diversified and physiologically relevant *in vitro* models^203^. Third, leveraging advanced multi-omics integration and artificial intelligence algorithms represents a promising strategy^132^. These tools are anticipated to enable the dynamic monitoring of senescence progression and facilitate the comprehensive construction of a spatiotemporal vascular aging atlas.

In conclusion, vascular aging is a prerequisite for the development of age-related diseases. A comprehensive understanding and timely intervention in vascular senescence are therefore of great importance. Future research should prioritize integrating multi-layered mechanisms and targeted vascular therapies. These integrated strategies are required to address the global challenges of aging and achieve healthy longevity.

## Figures and Tables

**Figure 1:**
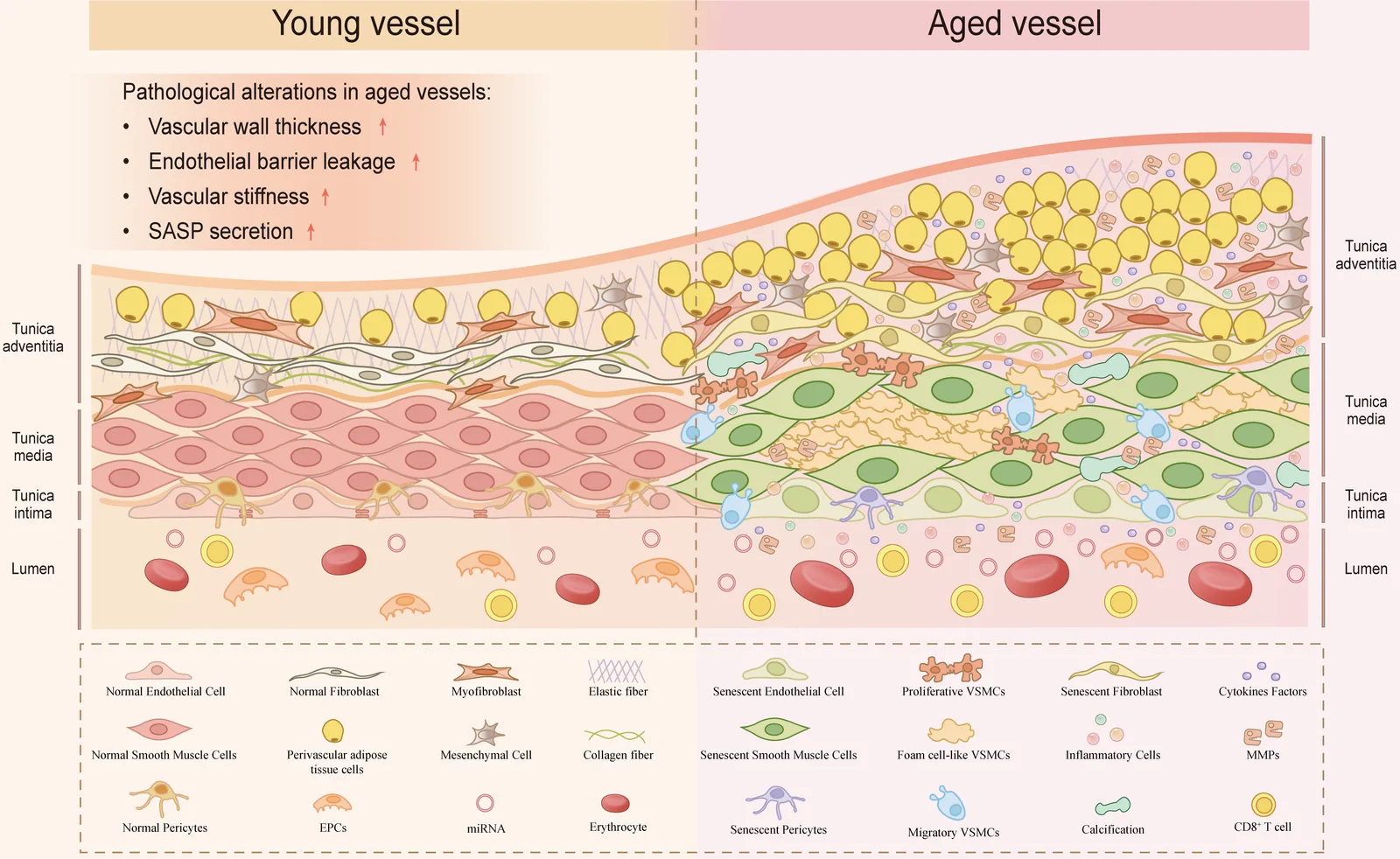
** Structural and cellular remodeling of the vasculature during aging.** Schematic comparison between young (left) and aged (right) blood vessels. Aging induces marked thickening of the vessel wall, accompanied by alterations in multiple cell types and ECM components. In the aged vasculature, ECs become senescent with impaired barrier function. VSMCs undergo phenotypic switching into proliferative, migratory, and foam-cell-like states, promoting calcification. Pericytes and fibroblasts also become senescent, while myofibroblasts accumulate. The ECM shows elastic fiber degeneration, excessive collagen deposition, and calcification. Inflammatory cells (including CD8^+^ T cells), cytokines, MMPs, and miRNAs are elevated, reflecting a pro-inflammatory SASP. Collectively, these integrated changes drive vascular stiffening, structural deterioration, and functional decay.

**Figure 2:**
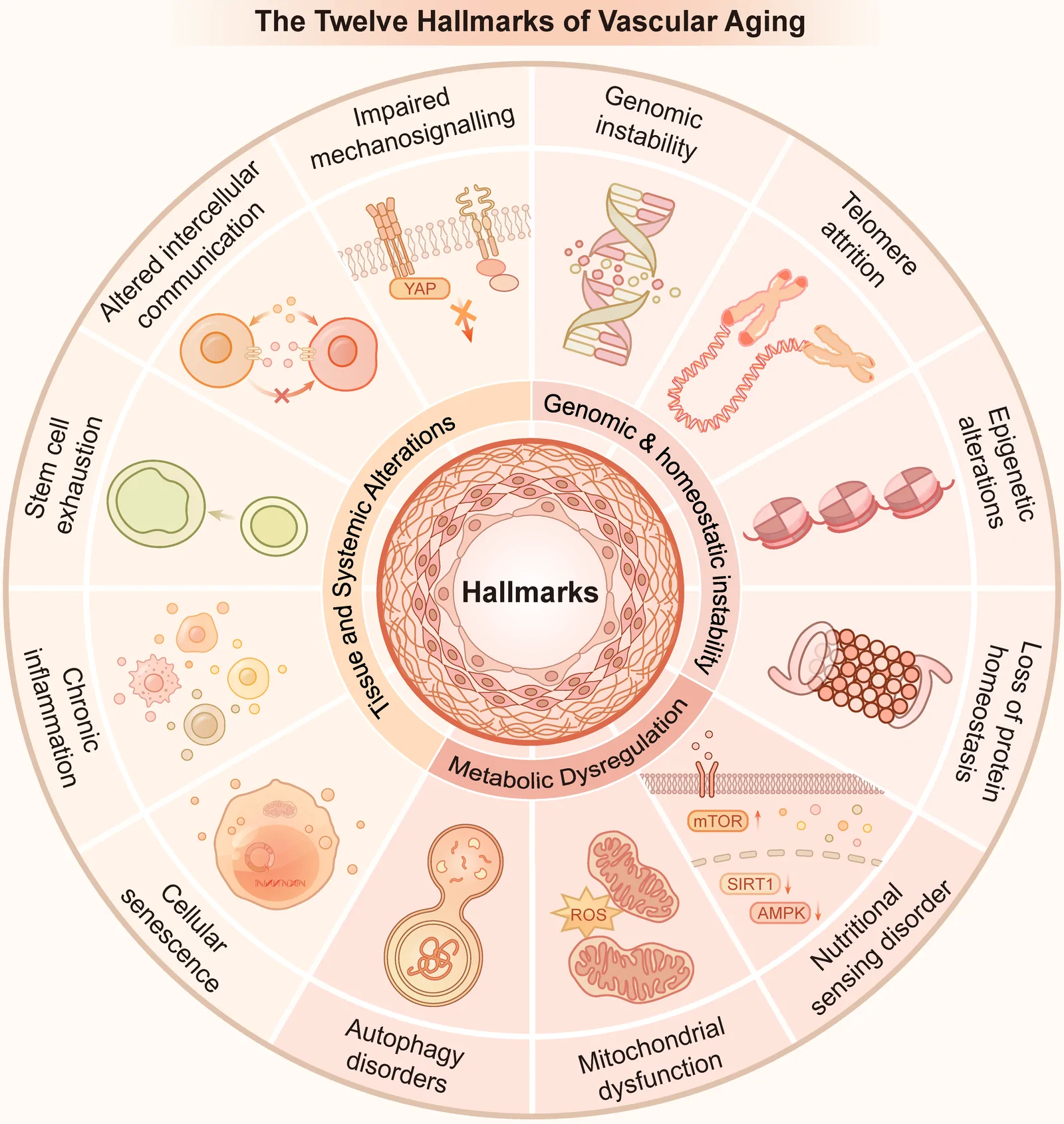
** The twelve hallmarks of vascular aging and their crosstalk.** Schematic illustrating the twelve hallmarks organized into three interconnected dimensions: (1) genomic and homeostatic instability (genomic instability, telomere attrition, epigenetic alterations, loss of protein homeostasis); (2) cellular and metabolic dysfunction (autophagy dysregulation, dysregulated nutrient sensing, mitochondrial dysfunction); and (3) cellular instability and microenvironmental evolution (cellular senescence, chronic inflammation, stem cell exhaustion, altered intercellular communication, impaired mechanosignaling). These multidimensional hallmarks collectively converge on the vascular wall, cooperatively driving the onset and progression of vascular aging.

**Figure 3:**
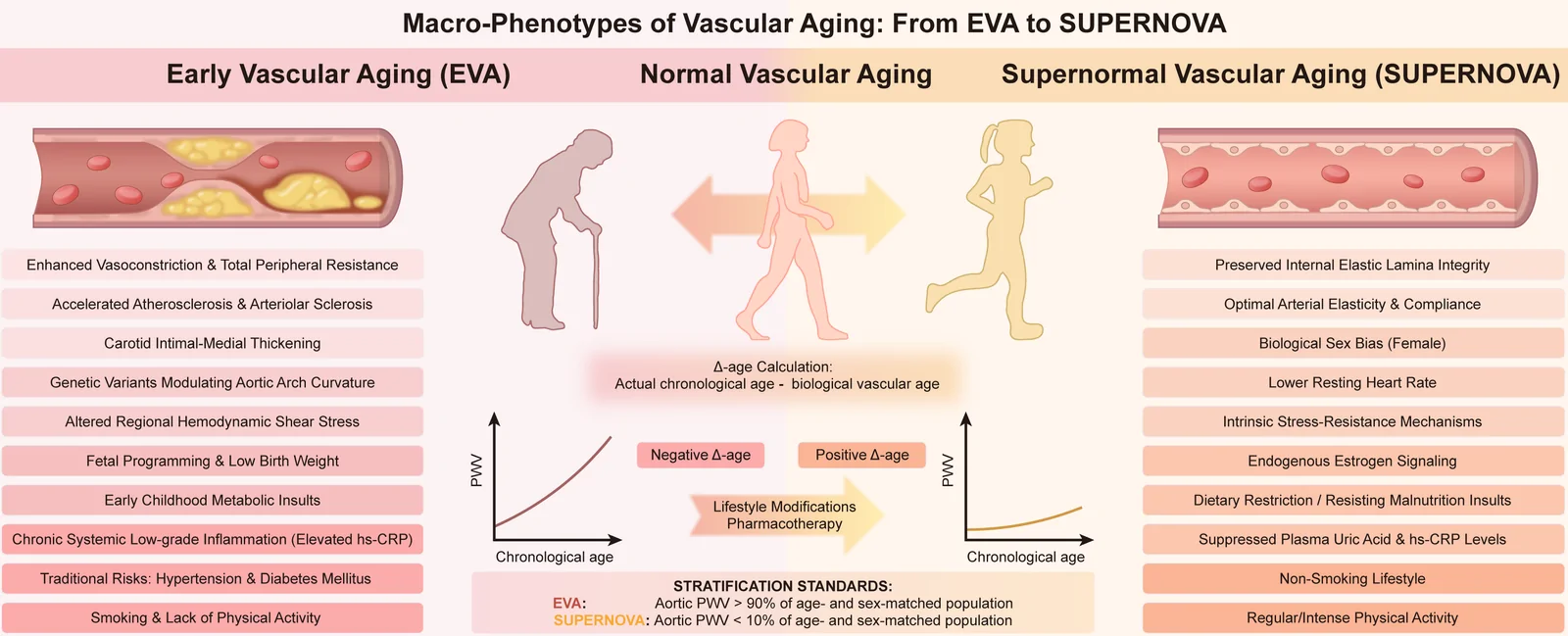
** Macro-phenotypes of vascular aging from EVA to SUPERNOVA.** Schematic illustration of the determinants and stratification standards for EVA and SUPERNOVA. The left panel lists the risk factors and structural alterations driving EVA. The right panel outlines the protective mechanisms and lifestyle factors contributing to SUPERNOVA. The central panel defines the aging trajectories based on PWV and Δ-age calculation.

**Figure 4:**
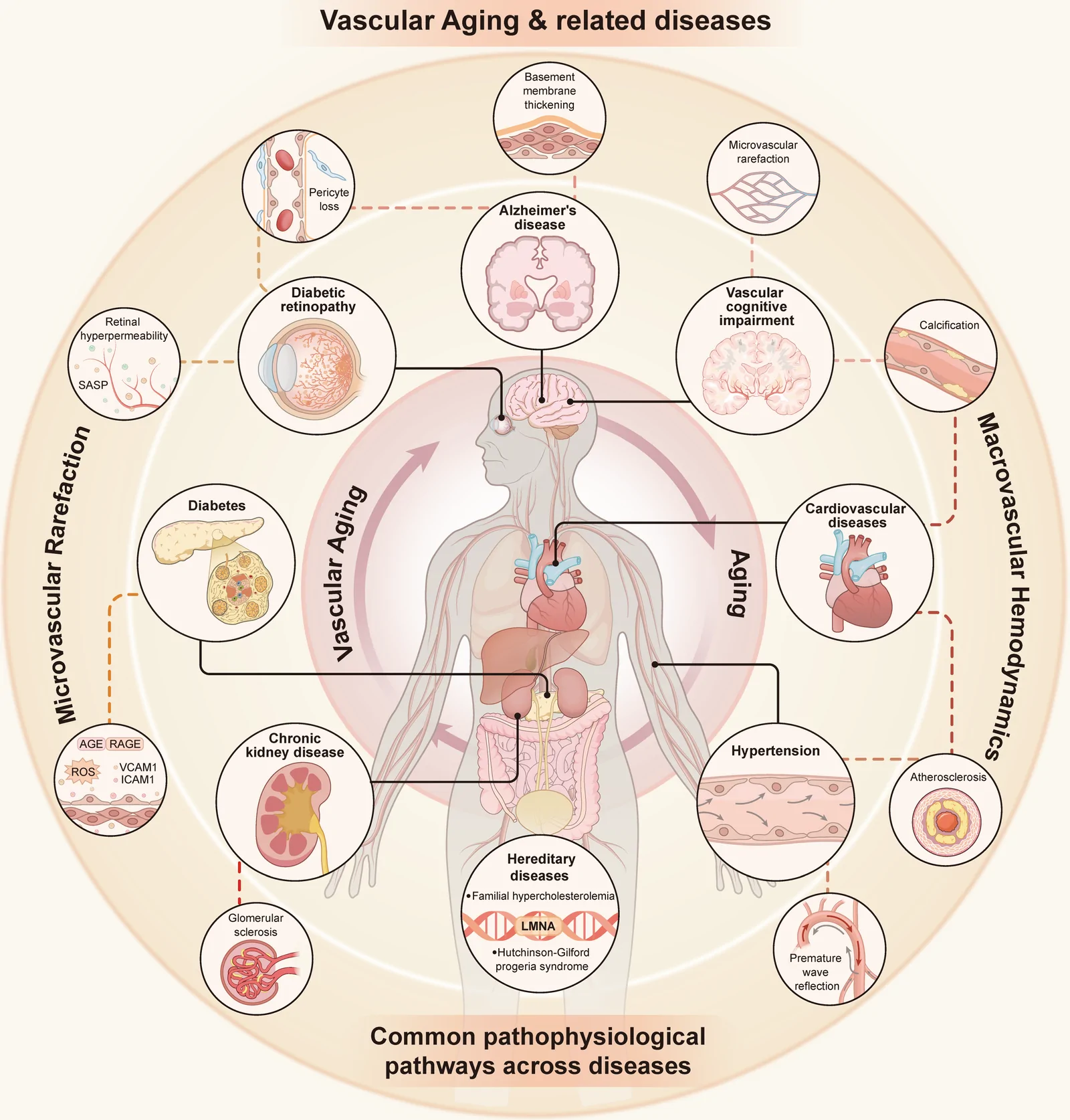
** Vascular aging as a common pathological driver of systemic diseases.** Schematic illustration of vascular aging and its associated systemic diseases. The inner rings depict age-related disorders across various organs, including AD, VCI, CVD, hypertension, CKD, diabetes, DR, alongside hereditary diseases. The outermost ring delineates the two primary shared pathophysiological dimensions—microvascular rarefaction and macrovascular hemodynamics—which serve as common pathways, driving specific organ pathologies through downstream microstructural alterations.

**Table 1:** Behavioral and pharmacological interventions to rescue vascular aging

Intervention strategies		Main effects	Side effects	References
Behavioral interventions	physical exercise and fitness	Reducing major mobility disability [Clinical]; Increasing blood volume by 20%-25% [Clinical]; Enhancing capillary density and mitochondrial content [Preclinical]; Protecting NO homeostasis and reducing ROS formation, improving vascular endothelial function [Preclinical]; Improving arterial stiffness and lowering of blood pressure [Clinical]; Reducing inflammatory markers [Preclinical].	Excessive exercise in the elderly may increase the risk of death; Excessive exercise may affect the structure and function of the heart and accelerate the aging of blood vessels.	141-145
	Calorie limitation	Increasing eNOS activity and improving vascular endothelial function [Preclinical]; Reducing age-enhancing oxidative stress [Preclinical]; Enhancing autophagy [Preclinical]; Reducing aortic elastin degradation [Clinical]; Improving systemic metabolism and limiting insulin resistance [Preclinical]; Upregulating SIRT1 expression and activity [Preclinical].	May result in decreased bone density and lean muscle mass; Triggers potential neurobehavioral or sleep quality fluctuations; Risk of malnutrition for young individuals.	146-150
	Mediterranean Diet	Reducing inflammatory markers [Preclinical]; Antioxidant [Preclinical]; Preventing atherosclerosis [Clinical]; Limiting telomere shortening [Preclinical]; Maintaining native vascular structure and function [Clinical].	May cause gastrointestinal discomfort due to poor digestive function; Imposes higher economic costs for long-term adherence.	151-154
Pharmacological interventions	Rapamycin and analogs	Delaying the progression of cardiac allograft vasculopathy [Clinical]; Alleviating arterial stiffness, blood pressure and left ventricular hypertrophy [Clinical]; Preventing coronary artery disease [Clinical]; Regulating vascular endothelium-mediated inflammation [Preclinical]; Inhibiting tumor angiogenesis and vascular permeability [Clinical].	Hypertriglyceridemia; Thrombocytopenia; Lymphopenia; Anemia.	165-167, 189
	Metformin	Ameliorating high glucose-induced oxidative stress [Preclinical]; Upregulating SIRT1 expression and activity [Preclinical]; Activating AMPK, downregulates mTOR, and diminishes SASP release [Preclinical]; Inhibiting endothelial senescence and improving endothelial function [Clinical]; Preventing diabetic cardiovascular complications and atherosclerosis [Clinical].	Mild gastrointestinal side effects; Hypoglycemia (when used with antidiabetic drugs or insulin); Lactic acidosis (in renal insufficiency; rare)	160-162, 190
	NAD precursor	Promoting NAD synthesis [Clinical]; Reducing oxidative stress in mitochondria [Preclinical]; Maintaining normal function of senescent vascular endothelium [Preclinical]; Restoring SIRT1/VEGF-dependent capillary density [Preclinical]; Rescuing neurovascular coupling response [Preclinical].	High-dose NAM acts as a pan-SIRT inhibitor and shortens lifespan in lower organisms; Long-term NAM improves healthy aging under high-fat stress.	172-177, 191, 192
	Resveratrol	Regulating retinal VEGF protein levels [Preclinical]; Reducing vascular senescent cells [Preclinical]; Inhibiting vascular leakage [Preclinical]; Reducing the loss of pericytes [Preclinical]; Decreasing the levels of pro-inflammatory proteins [Preclinical].	Mild to moderate gastrointestinal symptoms (at high dosage); Fails to modulate systemic glucose metabolism or insulin sensitivity in non-diabetic cohorts.	168-171, 193, 194
	Statins	Increasing NO levels in the endothelium [Preclinical]; Inhibiting the inflammatory response within the vessel wall [Preclinical]; Restricting NLRP3 activation and reducing atherogenesis [Preclinical]; Modulating GLP-1/adiponectin axis [Preclinical]; Lowering LDL cholesterol [Clinical].	Prolonged high dose may cause vascular calcification; Induces minor osteogenic trans-differentiation of VSMCs.	155-159, 195-199
	ACE & ARBs	Anti-Collagen Fibrosis [Clinical]; Enhancing endothelial function [Preclinical]; Mitigating arterial stiffness [Clinical]; Reducing vascular inflammation [Preclinical]	Intractable hypotension (under general anesthesia).	178-181, 200
	Probiotics & Prebiotics	Reshaping the gut microbiota [Preclinical]; Decreasing the level of TMAO [Preclinical]; Enhancing branched chain amino acids [Preclinical]; Preventing atherosclerosis [Preclinical].	Causing allergic reactions; Temporary constipation, flatulence, or stomach bloating.	182, 183, 201
	Senolytics	Removing senescent cells [Preclinical]; Disabling SCAPs [Preclinical]; Reducing capillary permeability [Preclinical]; Improving vasodilation and contraction [Clinical]	Potential to affect normal cell populations; Induces myelosuppression and neutropenia; Causes transient endothelial denudation within vessel walls.	184, 185, 202
